# Supplementary material for: Community social capital and the onset of functional disability among older adults in Japan: a multilevel longitudinal study using Japan Gerontological Evaluation Study (JAGES) data
Source: BMJ Open. 2019 Oct 8;9(10):e029279. doi: 10.1136/bmjopen-2019-029279 (PMC6797418; doi:10.1136/bmjopen-2019-029279)
Supplement: Author's manuscript [file bmjopen-2019-029279.draft_revisions.pdf]

BMJ Open is committed to open peer review. As part of this commitment we make the peer review history of every article we publish publicly available.

When an article is published we post the peer reviewers' comments and the authors' responses online. We also post the versions of the paper that were used during peer review. These are the versions that the peer review comments apply to.

The versions of the paper that follow are the versions that were submitted during the peer review process. They are not the versions of record or the final published versions. They should not be cited or distributed as the published version of this manuscript.

BMJ Open is an open access journal and the full, final, typeset and author-corrected version of record of the manuscript is available on our site with no access controls, subscription charges or pay-per-view fees (http://bmjopen.bmj.com).

If you have any questions on BMJ Open's open peer review process please email info.bmjopen@bmj.com

BMJ Open

Community social capital and the onset of functional disability among older adults in Japan: A multilevel longitudinal study using Japan Gerontological Evaluation Study (JAGES) data

Journal:	*BMJ Open*
Manuscript ID	bmjopen-2019-029279
Article Type:	Research
Date Submitted by the Author:	20-Jan-2019
Complete List of Authors:	Noguchi, Taiji; Nagoya City University, Department of Public Health<br>Kondo, Katsunori; Chiba University, Center for Preventive Medical Science<br>Saito, Masashige; Nihonfukushi University, Faculty of Social Welfare<br>Nakagawa-Senda, Hiroko; Nagoya City University, Department of Public Health<br>Suzuki, Sadao; Nagoya City University, Department of Public Helath
Keywords:	PUBLIC HEALTH, EPIDEMIOLOGY, GERIATRIC MEDICINE, SOCIAL MEDICINE

SCHOLARONE™
Manuscripts

Community social capital and the onset of functional disability among older adults in Japan: A multilevel longitudinal study using Japan Gerontological Evaluation Study (JAGES) data

Taiji Noguchi[1], Katsunori Kondo[2,3], Masashige Saito[4], Hiroko Nakagawa-Senda[1], Sadao Suzuki[1]

Author affiliations

[1]Department of Public Health, Nagoya City University Graduate School of Medical Sciences, Aichi, Japan

[2]Center for Preventive Medical Sciences, Chiba University, Chiba, Japan

[3]Center for Gerontology and Social Science, National Center for Geriatrics and Gerontology, Aichi, Japan

[4]Department of Social Welfare, Nihon Fukushi University, Aichi, Japan

Corresponding author:

Sadao Suzuki, MD, PhD

Department of Public Health

For peer review only

Nagoya City University Graduate School of Medical Sciences

1 Kawasumi, Mizuho-cho, Mizuho-ku, Nagoya, Aichi 467-8601, Japan

e-mail: ssuzuki@med.nagoya-cu.ac.jp

Tel: +81-52-853-8176, +81-52-853-8177

Fax : +81-52-842-3830

For peer review only

Abstract (256 words)

Objective:

The present study examined the association between community social capital and the onset of functional disability among older Japanese people by using validated indicators of social capital and a prospective multilevel analysis design.

Design:

Prospective cohort study

Setting:

We utilized data from the Japan Gerontological Evaluation Study (JAGES), established from August 2010 to January 2012 in 323 districts.

Participants:

The target population was restricted to non-institutionalized people aged 65 years or

older who were independent in activities of daily living. Participants included 73,021 people (34,051 men and 38,970 women) who were followed up over a 3-year period.

Primary outcome measure:

The primary outcome measure was the onset of functional disability, defined as a new registration in public long-term care insurance (LTCI) system records with a care-needs level of two or above, analysed with multilevel Cox proportional hazards regression models by community social capital (civic participation, social cohesion, and reciprocity).

Results:

The mean age of participants was 73.3 years (SD = 6.0) for men and 73.8 years (SD = 6.2) for women. During the study period, the onset of functional disability occurred in 1465 (4.3%) men and 1519 (3.9%) women. Of three community social capital variables, social cohesion significantly reduced the risk of onset of functional disability (hazard ratio = 0.910; 95% CI: 0.830–0.998) among men, after adjusting for individual social and behavioral variables. There was no significant effect among women.

Conclusions:

Living in a community with rich social cohesion is associated with a lower incidence of onset of functional disability among older Japanese men.

For peer review only - http://bmjopen.bmj.com/site/about/guidelines.xhtml

Strengths and limitations of this study

- This is the first prospective cohort study to examine the association between community social capital and the onset of functional disability among older people, by a large, nationwide population-based Japanese sample.
- To measure community social capital, an indicator consisting of validated multidimentional items was used, and we assessed there components of community social capital (civic participation, social cohesion, and reciprocioty).
- Multilevel survival analysis was used to examine community contextual characteristics for the onset of functional disability.
- More than 73,000 people aged 65 years or older participated and be followed up for 3 years period.
- While this study was a large sample size, the measurements were self-reported data.

Word count: 3,023 words

INTRODUCTION

In almost every country, the proportion of older people is growing at an increasing rate,[1] and Japan has displayed the fastest growth. In 2012, 32% of the Japanese

For peer review only - http://bmjopen.bmj.com/site/about/guidelines.xhtml

population was aged over 60 years, and this is expected to rise to 42% by 2050.[2] Age-related functional disability, defined as difficulty performing activities of daily living, is a very important public health issue worldwide.[3, 4] Because functional disability affects health status and the costs of long-term care,[4] the prevention of functional disability among older people is increasingly important.

Recently, there have been great efforts to research the effect of social capital on health.[5-13] Putnam defines social capital as "features of social organization, such trust, norms and networks that can improve the efficiency of society by facilitating coordinated actions."[5] There is considerable evidence of associations between social capital and various health indicators.[6-12] Although both ecologic and individual-level studies of social capital have yielded important insight, an appropriate examination of social capital as a collective (and contextual) influence on health requires multilevel analysis.[13] Moreover, to establish a causal relationship between social capital and health, observational studies must include prospective longitudinal analyses.[7] Several multilevel prospective studies have suggested contextual effects of social capital on health outcomes, mortality,[14, 15] self-rated health,[16, 17] suicide,[18] depression,[19, 20] and oral health.[21] Although two studies have reported an association between community social capital and the incidence of onset of functional disability among older

For peer review only - http://bmjopen.bmj.com/site/about/guidelines.xhtml

people,[22, 23] evidence remains insufficient. The study areas in this previous work were limited to certain parts of Japan, which limits the generalization of the results. Additionally, these studies' measures of community social capital might not provide a full picture of social capital because the scales used might fail to capture the multiple dimensions of social capital, such as its cognitive and structural aspects.[24, 25]

In the present study, we sought to examine the association between community social capital and the onset of functional disability among older people, using a prospective multilevel design and analyzing data from a nationwide survey in Japan. We measured community social capital using an indicator consisting of recently developed and validated multidimensional items.[26]

METHODS

Study population

We utilized the Japan Gerontological Evaluation Study (JAGES) 2010–2013 cohort data.[27] Baseline data were collected using a self-administered questionnaire survey conducted from August 2010 to January 2012 among 85,161 people aged ≥ 65 years. The sample was restricted to people who did not already have functional disabilities, where functional disability was defined as being certified as eligible to receive long-term public

For peer review only

For peer review only - http://bmjopen.bmj.com/site/about/guidelines.xhtml

care insurance (LTCI) system services. A simple random sample was obtained from the official residence registers in 13 large municipalities, and a complete census was taken of older residents residing in the remaining 11 smaller municipalities (response rate = 66.3%).

Survey data from 81,980 respondents who provided information for identification by the public LTCI system were linked to the public LTCI records dataset over a 3-year follow-up period beginning April 1, 2010. We excluded 4,549 respondents from 123 community areas with < 50 respondents to avoid non-precise values from small sample sizes,[26] 253 respondents with unknown areas of residence, 1,599 respondents who did not apply for public LTCI certification despite having basic activities of daily living (BADL) limitations, and, to avoid the problem of reverse causation, 2,558 respondents who did not complete the BADL items. Finally, we used data from 73,021 respondents in 323 community areas.

Measurements

Outcome

We collected information on the onset of functional disability from municipality-administered public LTCI records. The public LTCI system classifies frail older adults

For peer review only - http://bmjopen.bmj.com/site/about/guidelines.xhtml

into seven levels ("support need levels" 1 and 2 and "care need levels" 1–5; larger numbers indicate more severe need) using a nationally standardized and validated algorithm. This level is determined according to older adults' physical and mental care needs, regardless of informal care received,[28] and it is assessed both through computer-based and home-visit interviews with a trained health care professional and through examinations conducted by a primary physician.[29] In the computer-based assessment, time needed for care is calculated according to nine categories of care needs, including five BADL domains (bathing, eating, toileting, dressing, and transferring), assistance with instrumental activities of daily living (IADL), behavioral problems, rehabilitation, and medical services.[28] In our study, the onset of functional disability was defined as a new registration in the public LTCI records with a care-needs level of two or above, which requires at least 50 minutes of care daily and generally corresponds to needing any type of BADL care.[29] We used this outcome measurement because it has been found to reflect healthy life expectancy.[30]

Community social capital

To measure community social capital, individual-level baseline data were aggregated for each of the 323 local districts. We assessed three components of community social capital

For peer review only

For peer review only - http://bmjopen.bmj.com/site/about/guidelines.xhtml

(community civic participation, social cohesion, and reciprocity), based on the instrument Saito et al. developed and validated for measuring health-related community social capital.[27] Briefly, the community level was defined as the school district, a measure of community social capital was generated using factor analysis, and the factor scores for each small district were used as community social capital variables.[22, 27] Level of community civic participation was assessed by summing the percentages of participation in volunteer, sports, and hobby groups in each community. Level of community social cohesion was measured by summing the percentages answering “very” or “moderately” (with possible response categories of “very,” ”moderately,” “neutral” “disagree a little,” and “disagree”) to three items: trust (“Do you think people living in your area can be trusted, in general?”), perception of others’ intention to help (“Do you think people living in your area try to help others in most situations?”), and attachment to the residential area (“How attached are you to the area where you live?”). Level of community reciprocity was measured by summing the percentages answering “yes” to three items: receives emotional support (“Do you have someone who listens to your concerns and complaints?”), provides emotional support (“Do you listen to someone’s concerns and complaints?”), and receives instrumental support (“Do you have someone who looks after you when you are sick and confined to a bed for a few days?”). Community civic participation, social

For peer review only - http://bmjopen.bmj.com/site/about/guidelines.xhtml

cohesion, and reciprocity scores were standardized (subtracted from the mean and divided by the standard deviation). We applied school districts as the community unit because this was the smallest feasible area unit identifiable in the JAGES data; civic activities are often conducted within each school district, and older people can easily travel by foot or bicycle within the school district where they live.

Individual responses on community social capital indicators

The individual-level social components used, which are closely related to the components of community social capital, were group participation in the community, perception of community social capital, social support, and social isolation. Group participation in the community was measured as a count of participation in the following types of groups: volunteer, sports, or hobby groups. Perception of community social cohesion was measured as a count of a study participant's responses of "very" or "moderately" to the following items: trust, perception of others' intention to help, and attachment to the residential area. Social support was measured as a count of the number of the following types of social support each participant had: received emotional support, provided emotional support, and received instrumental support. Social isolation was measured using the frequency of meeting with friends: A few times per year or less was considered

For peer review only - http://bmjopen.bmj.com/site/about/guidelines.xhtml

moderate isolation, and more than once per month was considered non-isolation.

Covariates

Sociodemographic characteristics and baseline health status were included in the analysis as covariates. These variables were age, equivalized income, educational attainment, marital status, self-rated health, self-reported body mass index (BMI), IADL, present illness, depression, lifestyle (smoking history, alcohol consumption, frequency of going outside), and individual social components. Age was categorized as 65–69, 70–74, 75–79, 80–84, or 85 years or older. Educational attainment was categorized as < 6, 6–9, 10–12, or ≥ 13 years. Equivalized income was categorized as low (< 1,990,000 JPY; 120 JPY = 1 USD), middle (2,000,000–3,990,000 JPY), or high (≥ 4,000,000 JPY). Marital status was categorized married, separated/divorced, or never married. Living arrangement was categorized as living with others or living alone. Self-rated health was measured using a single question: "What is your current health status?" with response options of "excellent," "good," "fair," and "poor." BMI was categorized as < 18.5, 18.5–24.9, or ≥ 25. IADL was assessed using a five-item subscale of the Tokyo Metropolitan Institute of Gerontology Higher Competence Scale.[32] We categorized those who had difficulty with at least one item as "with difficulty"; others were categorized as "without

For peer review only - http://bmjopen.bmj.com/site/about/guidelines.xhtml

difficulty." Present illness was measured using the following yes/no question: "Do you receive treatment now?" Depression was assessed using the 15-item Japanese version of the Geriatric Depression Scale (GDS),[32] with scores categorized as no depression (0–4 points), depressive tendency (5–9 points), or depression (≥ 10 points). Smoking history was categorized as non-smoking, quit before 5 years, quit within 4 years, or currently smoking. Alcohol consumption was categorized as never, past drinker, or current drinker. Frequency of going outside was categorized as almost every day, one to three times per week, or once or twice per month or less. Urbanization based on population density was categorized as urban (≥ 1500 people/km^2), suburban (1000–1500 people/ km^2), or rural (< 1000 people/ km^2).

Statistical analysis

The data included 73,021 individuals (first level) nested in 323 local districts (second level). The median number of subjects in each local district was 90 (25th and 75th percentile: 63 and 317). The multilevel analysis framework assumes that individual health outcomes are partly dependent on the districts where individuals live. Multilevel models estimate the variation in outcomes across districts (random effects) and the effects of community-level variables on the outcome, adjusting for individual

For peer review only

compositional characteristics (fixed effects). Multilevel survival analysis using Cox proportional hazards regression models with stratification by sex was applied to calculate the hazard ratio (HR) and 95% confidence interval (95% CI) for functional disability during the follow-up period. HRs were estimated for a 1 SD change in the percentages of community social capital variables. We used the following analysis models. First, the null model was used to assess whether the onset of functional disability varied across districts. Then, the effect of community social capital on the onset of functional disability was investigated, adjusting for age, educational attainment, equivalized income, marital status, living arrangements, BMI, self-rated health, present illness, IADL, alcohol consumption, smoking history, and urbanization (Model 1). GDS score and frequency of going outside were then included (Model 2). The final model also included social isolation, group participation in the community, social perception of community social capital, and social support (Model 3). Because there was frequently missing data on the covariates, a "missing" category was created for the analyses. The significance level was set at $p < 0.05$. We used R (Version 3.4.3 for Windows) for all of the statistical analyses. Random effects models were estimated using the "coxme" function (coxme package).[33]

For peer review only - http://bmjopen.bmj.com/site/about/guidelines.xhtml

Ethical issues

JAGES respondents were informed that participation in the study was voluntary and that completing and returning the self-administered questionnaire by mail indicated their consent to participate in the study. The ethics committee at Nihon Fukushi University approved the protocol and informed consent procedure for the present study (No. 10-05). This study conformed to the principles embodied in the Declaration of Helsinki.

Patient and public involvement

No patients were involved in the development of the research question, study design or data interpretation in this study.

RESULTS

Of the 73,021 respondents over the follow-up period (average = 2.7 years), 34,051 were men and 38,970 were women. The average age was 73.3 years (SD = 6.0) for men and 73.8 years (SD = 6.2) for women. During the follow-up period, 1465 (4.3%) new cases of functional disability occurred among men; among women, there were 1519 (3.9%) new cases of functional disability.

For peer review only - http://bmjopen.bmj.com/site/about/guidelines.xhtml

Table 1 shows the descriptive characteristics of the respondents at baseline. Respondents with onset of functional disability were more likely to be older, separated/divorced, presently ill, current or former drinkers, moderately socially isolated, and living in rural areas, and to have lower educational attainment, lower equivalized income, lower BMI, poor self-rated health, depression, IADL difficulties, lower frequency of going outside, no community group participation, and lower social support. These tendencies were almost identical for men and women.

For peer review only

For peer review only - http://bmjopen.bmj.com/site/about/guidelines.xhtml

Table1. Respondent characteristics

	Men (n=34051)			Women (n=38970)		
	n	%	Incidence rate per 1000 person-years	n	%	Incidence rate per 1000 person-years
**Age (years)**						
65-69	11352	34.8	4.7	12018	32.1	2.7
70-74	9751	29.9	9.9	10993	29.4	5.6
75-79	7272	22.3	21.1	8570	22.9	14.7
80-84	4045	12.4	33.8	5006	13.4	34.7
85 or older	1631	5.0	79.4	2383	6.4	79.1
**Educational attainment**						
<6	497	1.5	48.5	1211	3.1	59.4
6-9	14523	42.7	18.3	18903	48.5	14.0
10-12	10517	30.9	11.9	12272	31.5	10.4
≥13	6844	20.1	12.6	4332	11.1	9.7
Other or missing	1670	4.9	28.7	2252	5.8	25.6
**Equivalized income**						
Low	22521	69.1	14.7	22708	60.6	12.6
Middle	5653	17.3	13.0	5495	14.7	11.5
High	925	2.8	10.3	832	2.2	13.5
Missing	4952	15.2	27.1	9935	26.5	20.1
**Marital status**						
Married	28361	87.0	14.4	21903	58.5	8.6
Separated/divorced	3571	11.0	26.6	14132	37.7	22.0
Never married	444	1.4	9.8	815	2.2	20.9
Other or missing	1675	5.1	25.7	2120	5.7	21.8
**Living arrangements**						
Living with other	30211	92.7	15.3	31306	83.6	13.6
Living alone	2287	7.0	19.1	6041	16.1	17.0
Missing	1553	4.8	27.5	1623	4.3	19.7
**Body mass index**						
<18.5	1798	5.5	38.9	3098	8.3	26.0
18.5-24.9	22990	70.6	14.1	24947	66.6	11.7
≥25.0	7199	22.1	11.5	7896	21.1	11.7
Missing	2064	6.3	36.4	3029	8.1	32.6
**Self-rated health**						
Excellent	4208	12.4	6.8	4173	10.7	6.3
Good	22743	66.8	11.8	27015	69.3	11.3
Fair	5778	17.0	33.3	6351	16.3	27.7
Poor	1021	3.0	57.9	878	2.3	49.9
Missing	301	0.9	26.7	553	1.4	22.3
**Present illness**						
No	8391	25.8	9.7	8454	22.6	9.5
Yes	23171	71.1	17.8	26981	72.0	15.5
Missing	2489	7.6	22.4	3535	9.4	17.9
**Geriatric Depression Scale**						
No depression	21055	64.6	11.2	22164	59.2	10.1

For peer review only

For peer review only - http://bmjopen.bmj.com/site/about/guidelines.xhtml

Depressive tendency	6063	18.6	22.3	6491	17.3	19.9
Depression	2013	6.2	29.3	2083	5.6	29.2
Missing	4920	15.1	24.7	8232	22.0	18.1
**Instrumental activities of daily living**						
Without difficulty	22452	68.9	10.8	31079	83.0	7.8
With difficulty	8996	27.6	27.4	5188	13.9	49.5
Missing	2603	8.0	23.8	2703	7.2	25.2
**Alcohol consumption**						
Never	18187	53.4	11.6	5405	13.9	7.8
Past	2022	5.9	27.2	366	0.9	21.5
Current	11710	34.4	20.5	30709	78.8	15.1
Missing	2132	6.3	20.6	2490	6.4	18.4
**Smoking history**						
Non-smoking	8191	25.1	14.4	31089	83.0	13.6
Non-smoking now, quit before 5 years	13967	42.9	16.0	1232	3.3	16.4
Non-smoking now, quit within 4 years	2934	9.0	16.6	446	1.2	12.8
Smoking	6305	19.3	16.5	1130	3.0	14.9
Missing	2654	8.1	20.8	5073	13.5	18.7
**Frequency of going outside**						
Once to twice a month or less	1905	5.6	48.5	2758	7.1	40.8
One to three times a week	10397	30.5	19.7	16099	41.3	16.2
Almost everyday	19632	57.7	10.6	17799	45.7	8.1
Missing	2117	6.2	22.8	2314	5.9	20.1
**Social isolation**						
Non-isolation	21650	66.4	12.7	29475	78.7	11.8
Moderately isolation	10038	30.8	20.7	6261	16.7	21.4
Missing	2363	7.3	28.6	3234	8.6	25.1
**Group participation in the community**						
Non	16011	47.0	18.3	14364	36.9	19.7
One	5562	16.3	9.3	6376	16.4	6.8
Over two	5180	15.2	7.9	6412	16.5	5.4
Missing	7298	21.4	22.8	11818	30.3	17.1
**Social support**						
Non	617	1.8	17.0	305	0.8	21.3
One	1260	3.7	17.8	690	1.8	21.3
Over two	30004	88.1	15.3	35523	91.2	13.4
Missing	2170	6.4	26.7	2452	6.3	25.5
**Perception of community social cohesion**						
Non	3312	9.7	18.4	4345	11.1	14.3
One	5001	14.7	13.7	6205	15.9	12.1
Over two	23560	69.2	15.1	25592	65.7	14.1
Missing	2178	6.4	29.5	2828	7.3	22.1
**Urbanization**						

For peer review only

Rural	10594	32.5	18.0	13169	35.2	16.2
Suburban	18560	57.0	15.8	20278	54.1	14.0
Urban	4897	15.0	11.9	5523	14.7	10.3

261

For peer review only

For peer review only - http://bmjopen.bmj.com/site/about/guidelines.xhtml

Tables 2 and 3 show the results of multilevel the survival analyses for men and women, respectively. In the multivariable-adjusted model (Model 1), among men, a significant association was observed between community-level social capital and incidence of functional disability for “social cohesion” (HR = 0.904, 95% CI: 0.824–0.992, p-value < 0.05). This association was maintained after adding individual GDS score and frequency of going outside (HR = 0.909, 95% CI: 0.829–0.996, p-value < 0.05; Model 2) and individual responses on community social capital indicators (HR = 0.910, 95% CI: 0.830–0.998; Model 3). Although the associations of incidence of functional disability with community-level civic participation and with reciprocity were not statistically significant, the point estimates for these effects were in the same direction, with HRs < 1.0 (civic participation: HR = 0.972, 95% CI: 0.893–1.058; reciprocity: HR = 0.920, 95% CI: 0.829–1.021; Model 3). Among women, no significant association was observed (civic participation: HR = 0.999, 95% CI: 0.918–1.087; social cohesion: HR = 0.930, 95% CI: 0.847–1.020; reciprocity: HR = 1.002, 95% CI: 0.901–1.114; Model 3).

For peer review only - http://bmjopen.bmj.com/site/about/guidelines.xhtml

Table 2. Multilevel survival analysis of functional disability among male respondents (n = 34,051)

	Model 1	Model 2	Model 3
	HR (95 % CI)	HR (95 % CI)	HR (95 % CI)
Fixed effects			
Community-level variables			
Community-level social capital			
Civic participation	0.956 (0.878-1.041)	0.959 (0.882-1.044)	0.972 (0.893-1.058)
Social cohesion	0.904 (0.824-0.992) *	0.909 (0.829-0.996) *	0.910 (0.830-0.998) *
Reciprocity	0.930 (0.837-1.032)	0.933 (0.841-1.035)	0.920 (0.829-1.021)
Urbanization (ref; Urban)			
Suburban	1.238 (0.977-1.568)	1.230 (0.972-1.557)	1.241 (0.981-1.571)
Rural	1.278 (0.986-1.655)	1.232 (0.951-1.596)	1.251 (0.967-1.620)
Individual-level variables			
Age (ref; 65-69 years)			
70-74	1.963 (1.603-2.403) **	1.940 (1.584-2.376) **	1.948 (1.591-2.387) **
75-79	3.624 (2.989-4.394) **	3.550 (2.928-4.305) **	3.549 (2.924-4.307) **
80-84	5.115 (4.189-6.246) **	4.876 (3.990-5.958) **	4.800 (3.923-5.873) **
85 or older	11.241 (9.126-13.846) **	10.371 (8.407-12.795) **	10.011 (8.099-12.375) **
Educational attainment (ref; <6)			
6-9	0.717 (0.549-0.938) *	0.738 (0.564-0.965) *	0.738 (0.564-0.966) *
10-12	0.682 (0.513-0.906) **	0.720 (0.542-0.957) *	0.727 (0.547-0.967) *
≥13	0.745 (0.554-1.000)	0.781 (0.581-1.050)	0.793 (0.590-1.067)
Equivalized income (ref; Low)			
Middle	1.234 (1.043-1.459) *	1.061 (0.903-1.247)	1.067 (0.908-1.254)
High	0.961 (0.523-1.769)	1.015 (0.673-1.530)	1.007 (0.668-1.519)
Marital status (ref; Married)			
Separated/divorced	1.028 (0.875-1.208)	1.218 (1.029-1.441) *	1.241 (1.048-1.470) *
Never married	0.962 (0.638-1.449)	0.907 (0.493-1.667)	0.929 (0.504-1.713)
Living arrangements (ref; Living with other)			
Living alone	1.012 (0.799-1.282	1.013 (0.798-1.284)	1.036 (0.815-1.317)
Body mass index (ref; 18.5-24.9)			
<18.5	1.688 (1.431-1.993) **	1.651 (1.399-1.949) **	1.633 (1.383-1.927) **
≥25.0	0.919 (0.792-1.065)	0.926 (0.798-1.074)	0.932 (0.804-1.081)
Self-rated health (ref; Excellent)			
Good	1.356 (1.068-1.722) *	1.289 (1.014-1.637) *	1.265 (0.996-1.608)
Fair	2.831 (2.205-3.636) **	2.464 (1.910-3.178) **	2.387 (1.850-3.079) **
Poor	4.396 (3.287-5.881) **	3.638 (2.699-4.903) **	3.515 (2.606-4.740) **
Present illness (ref; No)			
Yes	1.140 (0.978-1.327)	1.143 (0.981-1.331)	1.146 (0.983-1.334)
Instrumental activities of daily living (ref; Without difficulty)			
With difficulty	1.798 (1.606-2.013) **	1.654 (1.475-1.856) **	1.635 (1.457-1.835) **
Alcohol consumption (ref; Non)			
Past	1.089 (0.905-1.310)	1.074 (0.893-1.292)	1.055 (0.877-1.270)

For peer review only

Current	0.850 (0.757-0.954) **	0.866 (0.771-0.973) *	0.873 (0.777-0.981) *
**Smoking history (ref; Non-smoking)**			
Non-smoking now, quit before 5 years	1.089 (0.948-1.250)	1.075 (0.936-1.234)	1.071 (0.932-1.230)
Non-smoking now, quit within 4 years	1.256 (1.021-1.545) *	1.237 (1.006-1.522) *	1.221 (0.993-1.502)
Smoking	1.357 (1.153-1.598) **	1.320 (1.120-1.554) **	1.304 (1.107-1.536) **
**Geriatric Depression Scale (ref; No depression)**			
Depression tendency		1.224 (1.069-1.402) **	1.214 (1.059-1.392) **
Depression		1.222 (1.011-1.477) *	1.236 (1.017-1.501) *
**Frequency of going outside (ref; Almost everyday)**			
One to three times a week		1.237 (1.096-1.397) **	1.225 (1.084-1.383) **
Once to twice a month or less		1.899 (1.611-2.238) **	1.801 (1.524-2.128) **
**Social isolation (ref, Non-isolation)**			
Moderately isolation			1.060 (0.939-1.196)
**Group participation in the community (ref; Non)**			
One			0.778 (0.645-0.937) **
Over two			0.725 (0.588-0.894) **
**Social support (ref; Non)**			
One			0.993 (0.625-1.578)
Over two			1.287 (0.864-1.917)
**Perception of community social cohesion (ref; Non)**			
One			0.852 (0.686-1.057)
Over two			1.020 (0.853-1.220)
Random effects			
**Community-level variance**	0.0223	0.0210	0.0199

*$p < 0.05$, **$p < 0.01$

Values presented are HRs (hazard ratios) and 95% CIs (confidence intervals). Community-level social capital variables (civic participation, social cohesion, and reciprocity) are 1 SD increase estimates. Variance of the intercept in the null model = 0.0336

For peer review only - http://bmjopen.bmj.com/site/about/guidelines.xhtml

Table 3. Multilevel survival analysis of functional disability among female respondents (n = 38,970)

	Model 1	Model 2	Model 3
	HR (95% CI)	HR (95% CI)	HR (95% CI)
Fixed effects			
Community-level variables			
Community-level social capital			
Civic participation	0.986 (0.907-1.072)	0.987 (0.908-1.072)	0.999 (0.918-1.087)
Social cohesion	0.936 (0.854-1.027)	0.935 (0.853-1.025)	0.930 (0.847-1.020)
Reciprocity	1.003 (0.903-1.115)	1.007 (0.906-1.119)	1.002 (0.901-1.114)
Urbanization (ref; Urban)			
Suburban	0.977 (0.772-1.236)	0.968 (0.765-1.225)	0.978 (0.772-1.238)
Rural	1.045 (0.809-1.351)	1.013 (0.784-1.309)	1.024 (0.792-1.324)
Individual-level variables			
Age (ref; 65-69 years)			
70-74	1.911 (1.478-2.469) **	1.899 (1.469-2.455) **	1.896 (1.466-2.451) **
75-79	4.174 (3.293-5.290) **	4.111 (3.242-5.213) **	4.066 (3.203-5.161) **
80-84	8.084 (6.371-10.257) **	7.915 (6.232-10.052) **	7.733 (6.079-9.838) **
85 or older	14.654 (11.434-18.780) **	14.137 (11.016-18.143) **	13.749 (10.694-17.677) **
Educational attainment (ref; <6)			
6-9	0.723 (0.611-0.857) **	0.729 (0.615-0.863) **	0.734 (0.620-0.870) **
10-12	0.679 (0.561-0.822) **	0.687 (0.568-0.832) **	0.701 (0.578-0.849) **
≥13	0.792 (0.617-1.015)	0.806 (0.629-1.034)	0.826 (0.643-1.059)
Equivalized income (ref; Low)			
Middle	1.037 (0.876-1.229)	1.062 (0.896-1.259)	1.063 (0.897-1.261)
High	1.440 (0.989-2.097)	1.505 (1.033-2.193) *	1.500 (1.029-2.186) *
Marital status (ref; Married)			
Separated/divorced	1.078 (0.949-1.224)	1.068 (0.940-1.213)	1.064 (0.936-1.209)
Never married	1.356 (0.983-1.870)	1.330 (0.964-1.834)	1.338 (0.969-1.848)
Living arrangements (ref; Living with other)			
Living alone	1.055 (0.912-1.221)	1.044 (0.902-1.209)	1.071 (0.924-1.241)
Body mass index (ref; 18.5-24.9)			
<18.5	1.423 (1.220-1.661) **	1.395 (1.195-1.628) **	1.363 (1.168-1.592) **
≥25.0	0.997 (0.865-1.149)	0.994 (0.862-1.146)	1.003 (0.870-1.157)
Self-rated health (ref; Excellent)			
Good	1.404 (1.098-1.795) **	1.350 (1.054-1.727) *	1.333 (1.041-1.707) *
Fair	2.354 (1.818-3.049) **	2.111 (1.622-2.746) **	2.078 (1.596-2.706) **
Poor	3.439 (2.524-4.685) **	2.934 (2.138-4.027) **	2.856 (2.077-3.926) **
Present illness (ref; No)			
Yes	0.943 (0.811-1.097)	0.946 (0.813-1.101)	0.954 (0.820-1.111)
Instrumental activities of daily living (ref; Without difficulty)			
With difficulty	2.449 (2.167-2.767) **	2.287 (2.016-2.595) **	2.171 (1.911-2.467)
Alcohol consumption (ref; Non)			
Past	1.574 (1.005-2.467) *	1.550 (0.988-2.430)	1.624 (1.035-2.548) *

For peer review only

For peer review only - http://bmjopen.bmj.com/site/about/guidelines.xhtml

Current	0.915 (0.751-1.116)	0.936 (0.767-1.141)	0.949 (0.778-1.157)
**Smoking history (ref; Non-smoking)**			
Non-smoking now, quit before 5 years	1.490 (1.121-1.981) **	1.454 (1.093-1.934) *	1.455 (1.094-1.936) *
Non-smoking now, quit within 4 years	1.289 (0.758-2.193)	1.291 (0.759-2.197)	1.250 (0.735-2.129)
Smoking	1.468 (1.077-2.000) *	1.430 (1.048-1.949) *	1.400 (1.026-1.911) *
**Geriatric Depression Scale (ref; No depression)**			
Depression tendency		1.232 (1.074-1.414) **	1.240 (1.080-1.423) **
Depression		1.346 (1.116-1.624) **	1.364 (1.127-1.651) **
**Frequency of going outside (ref; Almost everyday)**			
One to three times a week		1.119 (0.985-1.272)	1.098 (0.965-1.249)
Once to twice a month or less		1.323 (1.120-1.563) **	1.257 (1.060-1.491) **
**Social isolation (ref, Non-isolation)**			
Moderately isolation			1.116 (0.977-1.275)
**Group participation in the community (ref; Non)**			
One			0.678 (0.555-0.829) **
Over two			0.736 (0.587-0.923) **
**Social support (ref; Non)**			
One			0.727 (0.408-1.295)
Over two			1.010 (0.619-1.647)
**Perception of community social cohesion (ref; Non)**			
One			0.912 (0.740-1.123)
Over two			1.145 (0.961-1.364)
Random effects			
**Community-level variance**	0.0202	0.0194	0.0209

$*p < 0.05$, $**p < 0.01$

Values presented are HRs (hazard ratios) and 95% CIs (confidence intervals). Community-level social capital variables (civic participation, social cohesion, and reciprocity) are 1 SD increase estimates. Variance of the intercept in the null model = 0.0351

For peer review only

For peer review only - http://bmjopen.bmj.com/site/about/guidelines.xhtml

DISCUSSION

To the best of our knowledge, this was the first study with a multilevel longitudinal design to examine the association between community social capital and the onset of functional disability using social capital indicators with verified validity in a large sample of older community-dwelling adults. The results suggested that living in a community with higher community social cohesion at baseline was associated with a lower future risk of functional disability, even after adjusting for individual responses on community social capital indicators. The present study indicated the importance of strategies to protect the health of older people through fostering cohesive communities with efforts such as promoting social connections and trust.

There are several possible pathways between community social cohesion and health. Social cohesion is determined by the resources available to members of tight-knit communities.[34] Cohesive communities might help residents to express trust toward their neighbors and to be psychologically healthier. Previous studies have revealed that neighborhood social cohesion positively affected older people's subjective well-being,[35, 36] and that cohesive communities prevented the occurrence of depressive symptoms in older people who lived alone and were at high risk of functional disability.[37] Thus, we considered high levels of community social cohesion to be potentially protective against

For peer review only - http://bmjopen.bmj.com/site/about/guidelines.xhtml

the onset of functional disability via the positive effects on psychological health, such as enhancing subjective well-being and inhibiting depressive symptoms.

Two previous studies examined the association between community social capital and the onset of functional disability in older people using multilevel longitudinal designs.[23, 24] Our results suggesting that higher community social cohesion was associated with lower risk of functional disability among men but not women were inconsistent with these previous studies. There are several possible reasons for this difference. First, both previous studies were surveys in a smaller area, compared with that in our research. Because our work used survey data from municipalities nationwide, the possibility for generalizing our findings might be higher. Second, the measurement index of community social capital in the previous studies differed from ours. Both previous studies used only one item ("general trust") to measure community social cohesion. In contrast, we used multidimensional indicators consisting of three measurement items with verified validity, which might be more accurate for examining community contextual effects. Therefore, our results might reflect more accurate estimates of the effects of community social cohesion on individual health.

In the present study, community social cohesion affected the onset of functional disability only among men. It is likely that, among people who are currently in the older

For peer review only - http://bmjopen.bmj.com/site/about/guidelines.xhtml

age groups, men sought and had stronger relationships with their colleagues before retirement than did women, given the nature of companies, particularly in Japan.[38] When this strong commitment is lost after retirement, these men may also experience a variety of changes in their living arrangements, leading to changes in their physical and mental health.[39] A cohesive community might be helpful in building new connections and encouraging social participation, which may keep men healthier and improve their psychological well-being. Honjo. et al. reported that rich social cohesion in a community buffered the risk of depression among older men living alone in Japan.[37] Thus, community cohesiveness may protect men's psychological health by helping them to build new connections in the community after retirement. However, further studies are needed to validate this hypothesis.

The present study had several strengths. First, using a large, nationwide population-based sample enabled us to conduct a community-level multilevel analysis to clarify the contextual relationship between community-level social capital and the onset of functional disability. Second, we used validated indicators consisting of multidimensional items to measure community social capital. Therefore, we may have appropriately captured the whole of community social capital. However, the study also had several limitations. First, because the measurement was based on a self-

For peer review only - http://bmjopen.bmj.com/site/about/guidelines.xhtml

administered questionnaire, the results are subject to response bias such as social desirability.[40] Social desirability bias may artificially inflate social capital as calculated from the responses to the questionnaire. Second, our study included no information about changes in social capital. Therefore, it is possible that unmeasured time-varying covariates such as economic changes or natural disasters may have biased our results. Third, although our study was a prospective cohort study, the follow-up period was moderately short. Considering the possibility of reverse causation, analyses with a longer follow-up period are necessary in the future.

CONCLUSION

In conclusion, this multilevel prospective cohort study found that higher levels of community social cohesion were associated with a lower incidence of onset of functional disability among older men, but not among older women, even after adjusting for individual social and behavioral variables. The findings suggest the importance of fostering cohesive communities to reduce the onset functional disability among older people.

ACKNOWLEDGMENTS

For peer review only - http://bmjopen.bmj.com/site/about/guidelines.xhtml

This study used data from the Japan Gerontological Evaluation Study (JAGES), conducted by the Center for Well-being and Society, Nihon Fukushi University, as one of their research projects. We thank Jennifer Barrett, PhD, from Edanz Group (www.edanzediting.com/ac) for editing a draft of this manuscript.

CONTRIBUTORS

TN had the idea for the study, participated in its design, performed the statistical analysis, and drafted the manuscript as the principal author. KK, the principal investigator of the JAGES project, helped to develop the idea of the study, participated in acquiring the data and in designing the study, and critically revised the manuscript. MS helped to develop the idea of the study, participated in acquiring the data and in designing the study, and critically revised the manuscript. HN helped with the data analysis and critically revised the manuscript. SS critically revised the manuscript. All authors read and approved the final manuscript.

FUNDING

This study used data from the Japan Gerontological Evaluation Study (JAGES), which was supported by the Ministry of Education, Culture, Sports, Science and Technology of

For peer review only

Japan's Supported Program for the Strategic Research Foundation at Private Universities (2009–2013), the Japan Society for the Promotion of Science KAKENHI (Grant Numbers JP18390200, JP22330172, JP22390400, JP23243070, JP23590786, JP23790710, JP24390469, JP24530698, JP24683018, JP25253052, JP25870573, JP25870881, JP26285138, JP26882010, JP15H01972), Health Labour Sciences Research Grants (H22-Choju-Shitei-008, H24-Junkanki [Seishu]-Ippan-007, H24-Chikyukibo-Ippan-009, H24-Choju-Wakate-009, H25-Kenki-Wakate-015, H25-Choju-Ippan-003, H26-Irryo-Shitei-003 [Fukkou], H26-Choju-Ippan-006, H27-Ninchisyou-Ippan-001), Research and Development 37 Grants for Longevity Science from the Japan Agency for Medical Research and Development (AMED), the Personal Health Record (PHR) Utilization Project from AMED, Research Funding for Longevity Sciences from the National Center for Geriatrics and Gerontology (24-17, 24-23, 29-42), and the World Health Organization Centre for Health Development (WHO Kobe Centre) (WHO APW 2017/713981). This work was supported by a grant from Nihon Fukushi University Research Institute for Health Sciences. The views and opinions expressed in this article are those of the authors and do not necessarily reflect the official policy or position of the funding organizations.

For peer review only

For peer review only - http://bmjopen.bmj.com/site/about/guidelines.xhtml

ETHICS APPROCAL

The ethics committee at Nihon Fukushi University approved the protocol and informed consent procedure for the present study (No. 10-05).

DATA SHARING

No additional data are available.

DATA AVAILABILITY STATEMENT

The data are from the JAGES study. All enquiries are to be addressed at the data management committee via e-mail: dataadmin.ml@jages.net. All JAGES datasets have ethical or legal restrictions for public deposition because of the inclusion of sensitive information about the human participants.

COMPERING INTERESTS

None declared.

REFERENCES

1 WHO. World Population Aging. New York: United Nations 2015.

For peer review only - http://bmjopen.bmj.com/site/about/guidelines.xhtml

2 International UaH. Aging in the Twenty-first Century: A Celebration and a Challenge. New York: UNFPA and HelpAge International 2012.

3 Ministry of Health, Labour and Welfare. Kaigo Hoken Jigyo Jyokyo Hokoku. Tokyo, Japan: Ministry of Health, Labour and Welfare 2010.

4 Keeler E, Guralnik JM, Tian H, et al. The impact of functional status on life expectancy in older persons. *J Gerontol A Biol Sci Med Sci* 2010;65A:727–33.

5 Putnam, RD. Making Democracy Work: Civic Traditions in Modern Italy. Princeton, NJ, USA: Princeton University Press 1993.

6 De Silva MJ, McKenzie K, Harpham T, et al. Social capital and mental illness: a systematic review. *J Epidemiol Community Health* 2005;59:619–27.

7 Murayama H, Fujiwara Y, Kawachi I. Social capital and health: a review of prospective multilevel studies. *J Epidemiol* 2012;22:179–87.

8 Story WT. Social capital and health in the least developed countries: a critical review of the literature and implications for a future research agenda. *Glob Public Health* 2013;8:983–99.

9 Nyqvist F, Forsman AK, Giuntoli G, et al. Social capital as a resource for mental well-being in older people: a systematic review. *Aging Ment Health* 2013;17:394–410.

10 McPherson KE, Kerr S, Morgan A, et al. The association between family and

For peer review only

For peer review only - http://bmjopen.bmj.com/site/about/guidelines.xhtml

community social capital and health risk behaviours in young people: an integrative review. *BMC Public Health* 2013;13:971.

11 Nyqvist F, Pape B, Pellfolk T, et al. Structural and cognitive aspects of social capital and all-cause mortality: a meta-analysis of cohort studies. *Soc Indic Res* 2014;116:545–66.

12 Choi M, Mesa-Frias M, Nuesch E, et al. Social capital, mortality, cardiovascular events and cancer: a systematic review of prospective studies. *Int J Epidemiol* 2014;43:1895–1920.

13 Kawachi I, Subramanian SV, Kim D. Social capital and health: a decade of progress and beyond. In: Kawachi I, Subramanian SV, Kim D, eds. Social Capital and Health. New York: Springer 2008:1–26.

14 Wen M, Cagney KA, Christakis NA. Effect of specific aspects of community social environment on the mortality of individuals diagnosed with serious illness. *Soc Sci Med* 2005;61:1119–34.

15 Mohan J, Twigg L, Barnard S, et al. Social capital, geography and health: a small-area analysis for England. *Soc Sci Med* 2005;60:1267–83.

16 Snelgrove JW, Pikhart H, Stafford M. A multilevel analysis of social capital and self-rated health: evidence from the British Household Panel Survey. *Soc Sci Med*

For peer review only

For peer review only - http://bmjopen.bmj.com/site/about/guidelines.xhtml

2009;68:1993–2001.

17 Oksanen T, Kouvonen A, Kivimäki M, et al. Social capital at work as a predictor of employee health: multilevel evidence from work units in Finland. *Soc Sci Med* 2008;66:637–49.

18 Desai RA, Dausey DJ, Rosenheck RA. Mental health service delivery and suicide risk: the role of individual patient and facility factors. *Am J Psychiatry* 2005;162:311–18.

19 Haseda M, Kondo N, Takagi D, et al. Community social capital and inequality in depressive symptoms among older Japanese adults: a multilevel study. *Health Place* 2018;52:8–17.

20 Yamaguchi M, Inoue Y, Shinozaki T, Saito M, Takagi D, Kondo K, Kondo N. Community social capital and depressive symptoms among older people in Japan: a multilevel longitudinal study. *J Epidemiol* (In press).

21 Koyama S, Aida J, Saito M, et al. Community social capital and tooth loss in Japanese older people: a longitudinal cohort study. *BMJ Open* 2016;6:e010768.

22 Aida J, Kondo K, Kawachi I, et al. Does social capital affect the incidence of functional disability in older Japanese? A prospective population-based cohort study. *J Epidemiol Community Health* 2013;67:42–7.

23 Imamura H, Hamano T, Michikawa T, et al. Relationships of community and

For peer review only

For peer review only - http://bmjopen.bmj.com/site/about/guidelines.xhtml

individual level social capital with activities of daily living and death by gender. *Int J Environ Res Public Health* 2016;13:860.

24 Harpham T, Grant E, Thomas E. Measuring social capital within health surveys: key issues. *Health Policy Plan* 2002;17:106–11.

25 Baum FE, Ziersch AM. Social capital. *J Epidemiol Community Health* 2003;57:320–3.

26 Saito M, Kondo N, Aida J, et al. Development of an instrument for community-level health related social capital among Japanese older people: the JAGES Project. *J Epidemiol* 2017;27:221–7.

27 Kondo K. Progress in aging epidemiology in Japan: the JAGES Project. *J Epidemiol* 2016;26:331–6.

28 Tsutsui T, Muramatsu N. Care-needs certification in the long-term care insurance system of Japan. *J Am Geriatr Soc* 2005;53:522–7.

29 Ministry of Health, Labour and Welfare. Long-term care, health and welfare services for the elderly. http://www.mhlw.go.jp/english/policy/care-welfare/care-welfare-elderly/index.html. Accessed 11 Nov 2018.

30 Matsuyama Y, Aida J, Watt RG, et al. Dental status and compression of life expectancy with disability. *J Dent Res* 2017;96:1006–13.

For peer review only - http://bmjopen.bmj.com/site/about/guidelines.xhtml

31 Koyano W, Shibata H, Nakazato K, et al. Measurement of competence: reliability and validity of the TMIG Index of Competence. *Arch Gerontol Geriatr* 1991;13:103–16.

32 Yesavage JA, Brink TL, Rose TL, et al. Development and validation of a geriatric depression screening scale: a preliminary report. *J Psychiatr Res* 1982;17:37–49.

33 Thabut G, Christie JD, Kremers WK, et al. Survival differences following lung transplantation among US transplant centers. *JAMA* 2010;304:53–60.

34 Kawachi I. Commentary: social capital and health: making the connections one step at a time. *Int J Epidemiol* 2006;35:989–93.

35 Cramm JM, Nieboer AP. Social cohesion and belonging predict the well-being of community-dwelling older people. *BMC Geriatr* 2015;15:30.

36 Cramm JM, van Dijk HM, Nieboer AP. The importance of neighborhood social cohesion and social capital for the well being of older adults in the community. *Tijdschr Gerontol Geriatr* 2013;44:50–8.

37 Honjo K, Tani Y, Saito M, et al. Living alone or with others and depressive symptoms, and effect modification by residential social cohesion among older adults in Japan: the JAGES Longitudinal Study. *J Epidemiol* 2018;28:315–22.

38 Suzuki E, Takao S, Subramanian SV, et al. Work-based social networks and health status among Japanese employees. *J Epidemiol Community Health* 2009;63:692–6.

For peer review only - http://bmjopen.bmj.com/site/about/guidelines.xhtml

39 Oksanen T, Virtanen M. Health and retirement: a complex relationship. *Eur J Ageing* 2012;9:221–5.

40 Heitmann BL. Social desirability bias in dietary self-report may compromise the validity of dietary intake measures: implications for diet disease relationships. *Int J Epidemiol* 1996;25:222–5.

For peer review only

STROBE Statement—Checklist of items that should be included in reports of ***cohort studies***

	Item No	Recommendation	Page No
**Title and abstract**	1	(*a*) Indicate the study's design with a commonly used term in the title or the abstract	1
		(*b*) Provide in the abstract an informative and balanced summary of what was done and what was found	2-3
**Introduction**			
Background/rationale	2	Explain the scientific background and rationale for the investigation being reported	4-6
Objectives	3	State specific objectives, including any prespecified hypotheses	6
**Methods**			
Study design	4	Present key elements of study design early in the paper	6
Setting	5	Describe the setting, locations, and relevant dates, including periods of recruitment, exposure, follow-up, and data collection	6-7
Participants	6	(*a*) Give the eligibility criteria, and the sources and methods of selection of participants. Describe methods of follow-up	6-7
		(*b*) For matched studies, give matching criteria and number of exposed and unexposed	non
Variables	7	Clearly define all outcomes, exposures, predictors, potential confounders, and effect modifiers. Give diagnostic criteria, if applicable	7-11
Data sources/ measurement	8*	For each variable of interest, give sources of data and details of methods of assessment (measurement). Describe comparability of assessment methods if there is more than one group	7-11
Bias	9	Describe any efforts to address potential sources of bias	6-7
Study size	10	Explain how the study size was arrived at	7
Quantitative variables	11	Explain how quantitative variables were handled in the analyses. If applicable, describe which groupings were chosen and why	8-10
Statistical methods	12	(*a*) Describe all statistical methods, including those used to control for confounding	12-13
		(*b*) Describe any methods used to examine subgroups and interactions	non
		(*c*) Explain how missing data were addressed	13
		(*d*) If applicable, explain how loss to follow-up was addressed	non
		(*e*) Describe any sensitivity analyses	non
**Results**			
Participants	13*	(a) Report numbers of individuals at each stage of study—eg numbers potentially eligible, examined for eligibility, confirmed eligible, included in the study, completing follow-up, and analysed	14
		(b) Give reasons for non-participation at each stage	non
		(c) Consider use of a flow diagram	non
Descriptive data	14*	(a) Give characteristics of study participants (eg demographic, clinical, social) and information on exposures and potential confounders	14-18
		(b) Indicate number of participants with missing data for each variable of interest	16-18
		(c) Summarise follow-up time (eg, average and total amount)	14
Outcome data	15*	Report numbers of outcome events or summary measures over time	14

For peer review only

Main results	16	(*a*) Give unadjusted estimates and, if applicable, confounder-adjusted estimates and their precision (eg, 95% confidence interval). Make clear which confounders were adjusted for and why they were included	19-23
		(*b*) Report category boundaries when continuous variables were categorized	non
		(*c*) If relevant, consider translating estimates of relative risk into absolute risk for a meaningful time period	non
Other analyses	17	Report other analyses done—eg analyses of subgroups and interactions, and sensitivity analyses	non
**Discussion**			
Key results	18	Summarise key results with reference to study objectives	24
Limitations	19	Discuss limitations of the study, taking into account sources of potential bias or imprecision. Discuss both direction and magnitude of any potential bias	26-27
Interpretation	20	Give a cautious overall interpretation of results considering objectives, limitations, multiplicity of analyses, results from similar studies, and other relevant evidence	24-26
Generalisability	21	Discuss the generalisability (external validity) of the study results	25
**Other information**			
Funding	22	Give the source of funding and the role of the funders for the present study and, if applicable, for the original study on which the present article is based	28-29

*Give information separately for exposed and unexposed groups.

Note: An Explanation and Elaboration article discusses each checklist item and gives methodological background and published examples of transparent reporting. The STROBE checklist is best used in conjunction with this article (freely available on the Web sites of PLoS Medicine at http://www.plosmedicine.org/, Annals of Internal Medicine at http://www.annals.org/, and Epidemiology at http://www.epidem.com/). Information on the STROBE Initiative is available at http://www.strobe-statement.org.

For peer review only - http://bmjopen.bmj.com/site/about/guidelines.xhtml

For peer review only

BMJ Open

Community social capital and the onset of functional disability among older adults in Japan: A multilevel longitudinal study using Japan Gerontological Evaluation Study (JAGES) data

Journal:	*BMJ Open*
Manuscript ID	bmjopen-2019-029279.R1
Article Type:	Research
Date Submitted by the Author:	25-Jun-2019
Complete List of Authors:	Noguchi, Taiji; Nagoya City University, Department of Public Health; National Center for Geriatrics and Gerontology, Center for Gerontology and Social Science<br>Kondo, Katsunori; Chiba University, Center for Preventive Medical Science; National Center for Geriatrics and Gerontology, Center for Gerontology and Social Science<br>Saito, Masashige; Nihonfukushi University, Faculty of Social Welfare<br>Nakagawa-Senda, Hiroko; Nagoya City University, Department of Public Health<br>Suzuki, Sadao; Nagoya City University, Department of Public Helath
**Primary Subject Heading**:	Epidemiology
Secondary Subject Heading:	Public health, Geriatric medicine
Keywords:	PUBLIC HEALTH, EPIDEMIOLOGY, GERIATRIC MEDICINE, SOCIAL MEDICINE

SCHOLARONE™
Manuscripts

Community social capital and the onset of functional disability among older adults in Japan: A multilevel longitudinal study using Japan Gerontological Evaluation Study (JAGES) data

Taiji Noguchi[1,2], Katsunori Kondo[2,3], Masashige Saito[4], Hiroko Nakagawa-Senda[1], Sadao Suzuki[1]

Author affiliations

[1]Department of Public Health, Nagoya City University Graduate School of Medical Sciences, Aichi, Japan

[2]Center for Gerontology and Social Science, National Center for Geriatrics and Gerontology, Aichi, Japan

[3]Center for Preventive Medical Sciences, Chiba University, Chiba, Japan

[4]Department of Social Welfare, Nihon Fukushi University, Aichi, Japan

Corresponding author:

Sadao Suzuki, MD, PhD

Department of Public Health

For peer review only

For peer review only - http://bmjopen.bmj.com/site/about/guidelines.xhtml

Nagoya City University Graduate School of Medical Sciences

1 Kawasumi, Mizuho-cho, Mizuho-ku, Nagoya, Aichi 467-8601, Japan

e-mail: ssuzuki@med.nagoya-cu.ac.jp

Tel: +81-52-853-8176, +81-52-853-8177

Fax : +81-52-842-3830

Abstract (255 words)

Objective:

The present study examined the association between community social capital and the onset of functional disability among older Japanese people by using validated indicators of social capital and a prospective multilevel design.

Design:

Prospective cohort study

Setting:

We utilized data from the Japan Gerontological Evaluation Study (JAGES), established from August 2010 to January 2012 in 323 districts.

Participants:

The target population was restricted to non-institutionalized people aged 65 years or

For peer review only

For peer review only - http://bmjopen.bmj.com/site/about/guidelines.xhtml

older who were independent in activities of daily living. Participants included 73,021 people (34,051 men and 38,970 women) who were followed up over a 3-year period.

Primary outcome measure:

The primary outcome measure was the onset of functional disability, defined as a new registration in public long-term care insurance (LTCI) system records with a care-needs level of two or above, analysed with multilevel Cox proportional hazards regression models by community social capital (civic participation, social cohesion, and reciprocity).

Results:

The mean age of participants was 73.3 years (SD = 6.0) for men and 73.8 years (SD = 6.2) for women. During the study period, the onset of functional disability occurred in 1465 (4.3%) men and 1519 (3.9%) women. Of three community social capital variables, social cohesion significantly reduced the risk of onset of functional disability (hazard ratio = 0.910; 95% CI: 0.830–0.998) among men, after adjusting for individual social and behavioral variables. There was no significant effect among women.

Conclusions:

Living in a community with rich social cohesion is associated with a lower incidence of onset of functional disability among older Japanese men.

For peer review only

For peer review only - http://bmjopen.bmj.com/site/about/guidelines.xhtml

Strengths and limitations of this study

- This is the first prospective cohort study to examine the association between community social capital and the onset of functional disability among older people, by a large, nationwide population-based Japanese sample.
- To measure community social capital, an indicator consisting of validated multidimensional items was used, and we assessed three components of community social capital (civic participation, social cohesion, and reciprocity).
- Multilevel survival analysis was used to examine community contextual characteristics for the onset of functional disability.
- More than 73,000 people aged 65 years or older participated and were followed up for 3 years period.
- While this study was a large sample size, the measurements were self-reported data.

Word count: 3,465 words

INTRODUCTION

In almost every country, the proportion of older people is growing at an increasing rate,[1] and Japan has displayed the fastest growth. In 2012, 32% of the Japanese

For peer review only - http://bmjopen.bmj.com/site/about/guidelines.xhtml

population was aged over 60 years, and this is expected to rise to 42% by 2050.[2] Age-related functional disability, defined as difficulty performing activities of daily living, is a very important public health issue worldwide.[3, 4] Because functional disability affects health status and the costs of long-term care,[4] the prevention of functional disability among older people is increasingly important.

Recently, there have been great efforts to research the effect of social capital on health.[5-13] Putnam defines social capital as "features of social organization, such trust, norms and networks that can improve the efficiency of society by facilitating coordinated actions."[5] There is considerable evidence of associations between social capital and various health indicators.[6-12] Although both ecologic and individual-level studies of social capital have yielded important insight, an appropriate examination of social capital as a collective (and contextual) influence on health requires multilevel analysis.[13] Prospective study designs are useful for establishing a valid relationship between social capital and health.[7] Several multilevel prospective studies have suggested contextual effects of social capital on health outcomes, mortality,[14, 15] self-rated health,[16, 17] suicide,[18] depression,[19, 20] and oral health.[21] Although two studies have reported an association between community social capital and the incidence of onset of functional disability among older people,[22, 23] evidence remains insufficient.

For peer review only - http://bmjopen.bmj.com/site/about/guidelines.xhtml

The study areas in this previous work were limited to certain parts of Japan, which limits the generalization of the results. Additionally, these studies' measures of community social capital might not provide a full picture of social capital because the scales used might fail to capture the multiple dimensions of social capital, such as its cognitive and structural aspects. [24, 25]

In the present study, we sought to examine the association between community social capital and the onset of functional disability among older people, using a prospective multilevel design and analyzing data from a nationwide survey in Japan. We measured community social capital using an indicator consisting of recently developed and validated multidimensional items.[26]

METHODS

Study population

We utilized the Japan Gerontological Evaluation Study (JAGES) 2010–2013 cohort data.[27] Baseline data were collected using a self-administered questionnaire survey conducted from August 2010 to January 2012 among 85,161 people aged ≥ 65 years. The sample was restricted to people who did not already have functional disabilities, where functional disability was defined as being certified as eligible to receive long-term public

For peer review only - http://bmjopen.bmj.com/site/about/guidelines.xhtml

care insurance (LTCI) system services. A simple random sample was obtained from the official residence registers in 13 large municipalities, and a complete census was taken of older residents residing in the remaining 11 smaller municipalities (response rate = 66.3%).

Survey data from 81,980 respondents who provided information for identification by the public LTCI system were linked to the public LTCI records dataset over a 3-year follow-up period beginning April 1, 2010. We excluded 4,549 respondents from 123 community areas with < 50 respondents to avoid non-precise values from small sample sizes,[26] 253 respondents with unknown areas of residence, 1,599 respondents who did not apply for public LTCI certification despite having basic activities of daily living (BADL) limitations, and, to avoid the problem of reverse causation, 2,558 respondents who did not complete the BADL items. Finally, we used data from 73,021 respondents in 323 community areas (figure 1).

Measurements

Outcome

We collected information on the onset of functional disability from municipality-administered public LTCI records. The public LTCI system classifies frail older adults

For peer review only - http://bmjopen.bmj.com/site/about/guidelines.xhtml

into seven levels ("support need levels" 1 and 2 and "care need levels" 1–5; larger numbers indicate more severe need) using a nationally standardized and validated algorithm. This level is determined according to older adults' physical and mental care needs, regardless of informal care received,[28] and it is assessed both through computer-based and home-visit interviews with a trained health care professional and through examinations conducted by a primary physician.[29] In the computer-based assessment, time needed for care is calculated according to nine categories of care needs, including five BADL domains (bathing, eating, toileting, dressing, and transferring), assistance with instrumental activities of daily living (IADL), behavioral problems, rehabilitation, and medical services.[28] In our study, the onset of functional disability was defined as a new registration in the public LTCI records with a care-needs level of two or above, which requires at least 50 minutes of care daily and generally corresponds to needing any type of BADL care.[29] We used this outcome measurement because it has been found to reflect healthy life expectancy.[30]

Community social capital

To measure community social capital, individual-level baseline data were aggregated for each of the 323 local districts. We assessed three components of community social capital

For peer review only

For peer review only - http://bmjopen.bmj.com/site/about/guidelines.xhtml

(community civic participation, social cohesion, and reciprocity), based on the instrument Saito et al. developed and validated for measuring health-related community social capital.[26] Briefly, the community level was defined as the school district, a measure of community social capital was generated using factor analysis, and the factor scores for each small district were used as community social capital variables.[22, 26] Level of community civic participation was assessed by summing the percentages of participation in volunteer, sports, and hobby groups in each community. Level of community social cohesion was measured by summing the percentages answering "very" or "moderately" (with possible response categories of "very," "moderately," "neutral" "disagree a little," and "disagree") to three items: trust ("Do you think people living in your area can be trusted, in general?"), perception of others' intention to help ("Do you think people living in your area try to help others in most situations?"), and attachment to the residential area ("How attached are you to the area where you live?"). Level of community reciprocity was measured by summing the percentages answering "yes" to three items: receives emotional support ("Do you have someone who listens to your concerns and complaints?"), provides emotional support ("Do you listen to someone's concerns and complaints?"), and receives instrumental support ("Do you have someone who looks after you when you are sick and confined to a bed for a few days?"). Community civic participation, social

For peer review only - http://bmjopen.bmj.com/site/about/guidelines.xhtml

cohesion, and reciprocity scores were standardized (subtracted from the mean and divided by the standard deviation). We applied school districts as the community unit because this was the smallest feasible area unit identifiable in the JAGES data. School districts are likely to represent former 'villages,' which existed before repeated municipality mergers took place in the last few decades in Japan. Civic activities are often conducted within each school district, and older people can easily travel on foot or by bicycle within the school district where they live.

Individual responses on community social capital indicators

The individual-level social components used, which are closely related to the components of community social capital, were group participation in the community, perception of community social capital, social support, and social isolation. Group participation in the community was measured as a count of participation in the following types of groups: volunteer, sports, or hobby groups. Perception of community social cohesion was measured as a count of a study participant's responses of "very" or "moderately" to the following items: trust, perception of others' intention to help, and attachment to the residential area. Social support was measured as a count of the number of the following types of social support each participant had: received emotional support, provided

For peer review only

For peer review only - http://bmjopen.bmj.com/site/about/guidelines.xhtml

emotional support, and received instrumental support. Social isolation was measured using the frequency of meeting with friends: A few times per year or less was considered moderate isolation, and more than once per month was considered non-isolation.

Covariates

Sociodemographic characteristics and baseline health status were included in the analysis as covariates. These variables were age, equivalized income, educational attainment, marital status, self-rated health, self-reported body mass index (BMI), IADL, present illness, depression, lifestyle (smoking history, alcohol consumption, frequency of going outside), and individual social components. Age was categorized as 65–69, 70–74, 75–79, 80–84, or 85 years or older. Educational attainment was categorized as < 6, 6–9, 10–12, or ≥ 13 years. Euivalized income was calculated by dividing the income of each household by the square root of the household size (number of family members); these figures were then categorized as low (< 1,990,000 JPY; 120 JPY = 1 USD), middle (2,000,000–3,990,000 JPY), or high (≥ 4,000,000 JPY). We used this index as a measure of household economic status because it adjusts for household size. Marital status was categorized married, separated/divorced, or never married. Living arrangement was categorized as living with others or living alone. Self-rated health was measured using

For peer review only - http://bmjopen.bmj.com/site/about/guidelines.xhtml

a single question: "What is your current health status?" with response options of "excellent," "good," "fair," and "poor." BMI was categorized as < 18.5, 18.5–24.9, or ≥ 25. IADL was assessed using a five-item subscale of the Tokyo Metropolitan Institute of Gerontology Higher Competence Scale.[31] We categorized those who had difficulty with at least one item as "with difficulty"; others were categorized as "without difficulty." Present illness was measured using the following yes/no question: "Do you receive treatment now?" Depression was assessed using the 15-item Japanese version of the Geriatric Depression Scale (GDS),[32] with scores categorized as no depression (0–4 points), depressive tendency (5–9 points), or depression (≥ 10 points). Smoking history was categorized as non-smoking, quit before 5 years, quit within 4 years, or currently smoking. Alcohol consumption was categorized as never, past drinker, or current drinker. Frequency of going outside was categorized as almost every day, one to three times per week, or once or twice per month or less. Urbanization based on population density was categorized as urban (≥ 1500 people/km^2), suburban (1000–1500 people/ km^2), or rural (< 1000 people/ km^2).

Statistical analysis

The data included 73,021 individuals (first level) nested in 323 local districts (second

For peer review only - http://bmjopen.bmj.com/site/about/guidelines.xhtml

217 level). The median number of subjects in each local district was 90 (25th and 75th
218 percentile: 63 and 317). The multilevel analysis framework assumes that individual
219 health outcomes are partly dependent on the districts where individuals live. Multilevel
220 models estimate the variation in outcomes across districts (random effects) and the
221 effects of community-level variables on the outcome, adjusting for individual
222 compositional characteristics (fixed effects). Multilevel survival analysis using Cox
223 proportional hazards regression models with stratification by sex was applied to
224 calculate the hazard ratio (HR) and 95% confidence interval (95% CI) for functional
225 disability during the follow-up period. HRs were estimated for a 1 SD change in the
226 percentages of community social capital variables. We used the following analysis models.
227 First, the null model was used to assess whether the onset of functional disability varied
228 across districts. Then, the effect of community social capital on the onset of functional
229 disability was investigated, adjusting for age, educational attainment, equivalized
230 income, marital status, living arrangements, BMI, self-rated health, present illness,
231 IADL, alcohol consumption, smoking history, and urbanization (Model 1). GDS score and
232 frequency of going outside were then included (Model 2). The final model also included
233 social isolation, group participation in the community, social perception of community
234 social capital, and social support (Model 3). Because there was frequently missing data

For peer review only - http://bmjopen.bmj.com/site/about/guidelines.xhtml

on the covariates, a "missing" category was created for the analyses. The significance level was set at $p < 0.05$. We used R (Version 3.4.3 for Windows) for all of the statistical analyses. Random effects models were estimated using the "coxme" function (coxme package).[33]

Ethical issues

JAGES respondents were informed that participation in the study was voluntary and that completing and returning the self-administered questionnaire by mail indicated their consent to participate in the study. The ethics committee at Nihon Fukushi University approved the protocol and informed consent procedure for the present study (No. 10-05). This study conformed to the principles embodied in the Declaration of Helsinki.

Patient and public involvement

No patients were involved in the development of the research question, study design or data interpretation in this study.

RESULTS

For peer review only - http://bmjopen.bmj.com/site/about/guidelines.xhtml

Of the 73,021 respondents over the follow-up period (average = 2.7 years), 34,051 were men and 38,970 were women. The average age was 73.3 years (SD = 6.0) for men and 73.8 years (SD = 6.2) for women. During the follow-up period, 1465 (4.3%) new cases of functional disability occurred among men; among women, there were 1519 (3.9%) new cases of functional disability.

Table 1 shows the descriptive characteristics of the respondents at baseline. Respondents with onset of functional disability were more likely to be older, separated/divorced, presently ill, current or former drinkers, moderately socially isolated, and living in rural areas, and to have lower educational attainment, lower equivalized income, lower BMI, poor self-rated health, depression, IADL difficulties, lower frequency of going outside, no community group participation, and lower social support. These tendencies were almost identical for men and women.

For peer review only

Table1. Respondent characteristics

	Men (n=34051)			Women (n=38970)		
	n	%	Incidence rate per 1000 person-years	n	%	Incidence rate per 1000 person-years
**Age (years)**						
65-69	11352	34.8	4.7	12018	32.1	2.7
70-74	9751	29.9	9.9	10993	29.4	5.6
75-79	7272	22.3	21.1	8570	22.9	14.7
80-84	4045	12.4	33.8	5006	13.4	34.7
85 or older	1631	5.0	79.4	2383	6.4	79.1
**Educational attainment**						
<6	497	1.5	48.5	1211	3.1	59.4
6-9	14523	42.7	18.3	18903	48.5	14.0
10-12	10517	30.9	11.9	12272	31.5	10.4
≥13	6844	20.1	12.6	4332	11.1	9.7
Other or missing	1670	4.9	28.7	2252	5.8	25.6
**Equivalized income**						
Low	22521	69.1	14.7	22708	60.6	12.6
Middle	5653	17.3	13.0	5495	14.7	11.5
High	925	2.8	10.3	832	2.2	13.5
Missing	4952	15.2	27.1	9935	26.5	20.1
**Marital status**						
Married	28361	87.0	14.4	21903	58.5	8.6
Separated/divorced	3571	11.0	26.6	14132	37.7	22.0
Never married	444	1.4	9.8	815	2.2	20.9
Other or missing	1675	5.1	25.7	2120	5.7	21.8
**Living arrangements**						
Living with other	30211	92.7	15.3	31306	83.6	13.6
Living alone	2287	7.0	19.1	6041	16.1	17.0
Missing	1553	4.8	27.5	1623	4.3	19.7
**Body mass index**						
<18.5	1798	5.5	38.9	3098	8.3	26.0
18.5-24.9	22990	70.6	14.1	24947	66.6	11.7
≥25.0	7199	22.1	11.5	7896	21.1	11.7
Missing	2064	6.3	36.4	3029	8.1	32.6
**Self-rated health**						
Excellent	4208	12.4	6.8	4173	10.7	6.3
Good	22743	66.8	11.8	27015	69.3	11.3
Fair	5778	17.0	33.3	6351	16.3	27.7
Poor	1021	3.0	57.9	878	2.3	49.9
Missing	301	0.9	26.7	553	1.4	22.3
**Present illness**						
No	8391	25.8	9.7	8454	22.6	9.5
Yes	23171	71.1	17.8	26981	72.0	15.5
Missing	2489	7.6	22.4	3535	9.4	17.9
**Geriatric Depression Scale**						
No depression	21055	64.6	11.2	22164	59.2	10.1

For peer review only

Depressive tendency	6063	18.6	22.3	6491	17.3	19.9
Depression	2013	6.2	29.3	2083	5.6	29.2
Missing	4920	15.1	24.7	8232	22.0	18.1
**Instrumental activities of daily living**						
Without difficulty	22452	68.9	10.8	31079	83.0	7.8
With difficulty	8996	27.6	27.4	5188	13.9	49.5
Missing	2603	8.0	23.8	2703	7.2	25.2
**Alcohol consumption**						
Never	18187	53.4	11.6	5405	13.9	7.8
Past	2022	5.9	27.2	366	0.9	21.5
Current	11710	34.4	20.5	30709	78.8	15.1
Missing	2132	6.3	20.6	2490	6.4	18.4
**Smoking history**						
Non-smoking	8191	25.1	14.4	31089	83.0	13.6
Non-smoking now, quit before 5 years	13967	42.9	16.0	1232	3.3	16.4
Non-smoking now, quit within 4 years	2934	9.0	16.6	446	1.2	12.8
Smoking	6305	19.3	16.5	1130	3.0	14.9
Missing	2654	8.1	20.8	5073	13.5	18.7
**Frequency of going outside**						
Once to twice a month or less	1905	5.6	48.5	2758	7.1	40.8
One to three times a week	10397	30.5	19.7	16099	41.3	16.2
Almost everyday	19632	57.7	10.6	17799	45.7	8.1
Missing	2117	6.2	22.8	2314	5.9	20.1
**Social isolation**						
Non-isolation	21650	66.4	12.7	29475	78.7	11.8
Moderately isolation	10038	30.8	20.7	6261	16.7	21.4
Missing	2363	7.3	28.6	3234	8.6	25.1
**Group participation in the community**						
Non	16011	47.0	18.3	14364	36.9	19.7
One	5562	16.3	9.3	6376	16.4	6.8
Over two	5180	15.2	7.9	6412	16.5	5.4
Missing	7298	21.4	22.8	11818	30.3	17.1
**Social support**						
Non	617	1.8	17.0	305	0.8	21.3
One	1260	3.7	17.8	690	1.8	21.3
Over two	30004	88.1	15.3	35523	91.2	13.4
Missing	2170	6.4	26.7	2452	6.3	25.5
**Perception of community social cohesion**						
Non	3312	9.7	18.4	4345	11.1	14.3
One	5001	14.7	13.7	6205	15.9	12.1
Over two	23560	69.2	15.1	25592	65.7	14.1
Missing	2178	6.4	29.5	2828	7.3	22.1
**Urbanization**						

For peer review only

Rural	10594	32.5	18.0	13169	35.2	16.2
Suburban	18560	57.0	15.8	20278	54.1	14.0
Urban	4897	15.0	11.9	5523	14.7	10.3

For peer review only

Tables 2 and 3 show the results of multilevel the survival analyses for men and women, respectively. In the multivariable-adjusted model (Model 1), among men, a significant association was observed between community-level social capital and incidence of functional disability for “social cohesion” (HR = 0.904, 95% CI: 0.824–0.992, p-value < 0.05). This association was maintained after adding individual GDS score and frequency of going outside (HR = 0.909, 95% CI: 0.829–0.996, p-value < 0.05; Model 2) and individual responses on community social capital indicators (HR = 0.910, 95% CI: 0.830–0.998; Model 3). Although the associations of incidence of functional disability with community-level civic participation and with reciprocity were not statistically significant, the point estimates for these effects were in the same direction, with HRs < 1.0 (civic participation: HR = 0.972, 95% CI: 0.893–1.058; reciprocity: HR = 0.920, 95% CI: 0.829–1.021; Model 3). Among women, no significant association was observed (civic participation: HR = 0.999, 95% CI: 0.918–1.087; social cohesion: HR = 0.930, 95% CI: 0.847–1.020; reciprocity: HR = 1.002, 95% CI: 0.901–1.114; Model 3).

For peer review only

For peer review only - http://bmjopen.bmj.com/site/about/guidelines.xhtml

281 Table 2. Multilevel survival analysis of functional disability among male respondents (n = 34,051)

	Model 1	Model 2	Model 3
	HR (95 % CI)	HR (95 % CI)	HR (95 % CI)
Fixed effects			
Community-level variables			
Community-level social capital			
Civic participation	0.956 (0.878-1.041)	0.959 (0.882-1.044)	0.972 (0.893-1.058)
Social cohesion	0.904 (0.824-0.992) *	0.909 (0.829-0.996) *	0.910 (0.830-0.998) *
Reciprocity	0.930 (0.837-1.032)	0.933 (0.841-1.035)	0.920 (0.829-1.021)
Urbanization (ref; Urban)			
Suburban	1.238 (0.977-1.568)	1.230 (0.972-1.557)	1.241 (0.981-1.571)
Rural	1.278 (0.986-1.655)	1.232 (0.951-1.596)	1.251 (0.967-1.620)
Individual-level variables			
Age (ref; 65-69 years)			
70-74	1.963 (1.603-2.403) **	1.940 (1.584-2.376) **	1.948 (1.591-2.387) **
75-79	3.624 (2.989-4.394) **	3.550 (2.928-4.305) **	3.549 (2.924-4.307) **
80-84	5.115 (4.189-6.246) **	4.876 (3.990-5.958) **	4.800 (3.923-5.873) **
85 or older	11.241 (9.126-13.846) **	10.371 (8.407-12.795) **	10.011 (8.099-12.375) **
Educational attainment (ref; <6)			
6-9	0.717 (0.549-0.938) *	0.738 (0.564-0.965) *	0.738 (0.564-0.966) *
10-12	0.682 (0.513-0.906) **	0.720 (0.542-0.957) *	0.727 (0.547-0.967) *
≥13	0.745 (0.554-1.000)	0.781 (0.581-1.050)	0.793 (0.590-1.067)
Equivalized income (ref; Low)			
Middle	1.234 (1.043-1.459) *	1.061 (0.903-1.247)	1.067 (0.908-1.254)
High	0.961 (0.523-1.769)	1.015 (0.673-1.530)	1.007 (0.668-1.519)
Marital status (ref; Married)			
Separated/divorced	1.028 (0.875-1.208)	1.218 (1.029-1.441) *	1.241 (1.048-1.470) *
Never married	0.962 (0.638-1.449)	0.907 (0.493-1.667)	0.929 (0.504-1.713)
Living arrangements (ref; Living with other)			
Living alone	1.012 (0.799-1.282	1.013 (0.798-1.284)	1.036 (0.815-1.317)
Body mass index (ref; 18.5-24.9)			
<18.5	1.688 (1.431-1.993) **	1.651 (1.399-1.949) **	1.633 (1.383-1.927) **
≥25.0	0.919 (0.792-1.065)	0.926 (0.798-1.074)	0.932 (0.804-1.081)
Self-rated health (ref; Excellent)			
Good	1.356 (1.068-1.722) *	1.289 (1.014-1.637) *	1.265 (0.996-1.608)
Fair	2.831 (2.205-3.636) **	2.464 (1.910-3.178) **	2.387 (1.850-3.079) **
Poor	4.396 (3.287-5.881) **	3.638 (2.699-4.903) **	3.515 (2.606-4.740) **
Present illness (ref; No)			
Yes	1.140 (0.978-1.327)	1.143 (0.981-1.331)	1.146 (0.983-1.334)
Instrumental activities of daily living (ref; Without difficulty)			
With difficulty	1.798 (1.606-2.013) **	1.654 (1.475-1.856) **	1.635 (1.457-1.835) **
Alcohol consumption (ref; Non)			
Past	1.089 (0.905-1.310)	1.074 (0.893-1.292)	1.055 (0.877-1.270)

For peer review only - http://bmjopen.bmj.com/site/about/guidelines.xhtml

Current	0.850 (0.757-0.954) **	0.866 (0.771-0.973) *	0.873 (0.777-0.981) *
**Smoking history (ref; Non-smoking)**			
Non-smoking now, quit before 5 years	1.089 (0.948-1.250)	1.075 (0.936-1.234)	1.071 (0.932-1.230)
Non-smoking now, quit within 4 years	1.256 (1.021-1.545) *	1.237 (1.006-1.522) *	1.221 (0.993-1.502)
Smoking	1.357 (1.153-1.598) **	1.320 (1.120-1.554) **	1.304 (1.107-1.536) **
**Geriatric Depression Scale (ref; No depression)**			
Depression tendency		1.224 (1.069-1.402) **	1.214 (1.059-1.392) **
Depression		1.222 (1.011-1.477) *	1.236 (1.017-1.501) *
**Frequency of going outside (ref; Almost everyday)**			
One to three times a week		1.237 (1.096-1.397) **	1.225 (1.084-1.383) **
Once to twice a month or less		1.899 (1.611-2.238) **	1.801 (1.524-2.128) **
**Social isolation (ref, Non-isolation)**			
Moderately isolation			1.060 (0.939-1.196)
**Group participation in the community (ref; Non)**			
One			0.778 (0.645-0.937) **
Over two			0.725 (0.588-0.894) **
**Social support (ref; Non)**			
One			0.993 (0.625-1.578)
Over two			1.287 (0.864-1.917)
**Perception of community social cohesion (ref; Non)**			
One			0.852 (0.686-1.057)
Over two			1.020 (0.853-1.220)
Random effects			
**Community-level variance**	0.0223	0.0210	0.0199

*$p < 0.05$, **$p < 0.01$

Values presented are HRs (hazard ratios) and 95% CIs (confidence intervals). Community-level social capital variables (civic participation, social cohesion, and reciprocity) are 1 SD increase estimates. Variance of the intercept in the null model = 0.0336

For peer review only

For peer review only - http://bmjopen.bmj.com/site/about/guidelines.xhtml

Table 3. Multilevel survival analysis of functional disability among female respondents (n = 38,970)

	Model 1	Model 2	Model 3
	HR (95% CI)	HR (95% CI)	HR (95% CI)
Fixed effects			
Community-level variables			
Community-level social capital			
Civic participation	0.986 (0.907-1.072)	0.987 (0.908-1.072)	0.999 (0.918-1.087)
Social cohesion	0.936 (0.854-1.027)	0.935 (0.853-1.025)	0.930 (0.847-1.020)
Reciprocity	1.003 (0.903-1.115)	1.007 (0.906-1.119)	1.002 (0.901-1.114)
Urbanization (ref; Urban)			
Suburban	0.977 (0.772-1.236)	0.968 (0.765-1.225)	0.978 (0.772-1.238)
Rural	1.045 (0.809-1.351)	1.013 (0.784-1.309)	1.024 (0.792-1.324)
Individual-level variables			
Age (ref; 65-69 years)			
70-74	1.911 (1.478-2.469) **	1.899 (1.469-2.455) **	1.896 (1.466-2.451) **
75-79	4.174 (3.293-5.290) **	4.111 (3.242-5.213) **	4.066 (3.203-5.161) **
80-84	8.084 (6.371-10.257) **	7.915 (6.232-10.052) **	7.733 (6.079-9.838) **
85 or older	14.654 (11.434-18.780) **	14.137 (11.016-18.143) **	13.749 (10.694-17.677) **
Educational attainment (ref; <6)			
6-9	0.723 (0.611-0.857) **	0.729 (0.615-0.863) **	0.734 (0.620-0.870) **
10-12	0.679 (0.561-0.822) **	0.687 (0.568-0.832) **	0.701 (0.578-0.849) **
≥13	0.792 (0.617-1.015)	0.806 (0.629-1.034)	0.826 (0.643-1.059)
Equivalized income (ref; Low)			
Middle	1.037 (0.876-1.229)	1.062 (0.896-1.259)	1.063 (0.897-1.261)
High	1.440 (0.989-2.097)	1.505 (1.033-2.193) *	1.500 (1.029-2.186) *
Marital status (ref; Married)			
Separated/divorced	1.078 (0.949-1.224)	1.068 (0.940-1.213)	1.064 (0.936-1.209)
Never married	1.356 (0.983-1.870)	1.330 (0.964-1.834)	1.338 (0.969-1.848)
Living arrangements (ref; Living with other)			
Living alone	1.055 (0.912-1.221)	1.044 (0.902-1.209)	1.071 (0.924-1.241)
Body mass index (ref; 18.5-24.9)			
<18.5	1.423 (1.220-1.661) **	1.395 (1.195-1.628) **	1.363 (1.168-1.592) **
≥25.0	0.997 (0.865-1.149)	0.994 (0.862-1.146)	1.003 (0.870-1.157)
Self-rated health (ref; Excellent)			
Good	1.404 (1.098-1.795) **	1.350 (1.054-1.727) *	1.333 (1.041-1.707) *
Fair	2.354 (1.818-3.049) **	2.111 (1.622-2.746) **	2.078 (1.596-2.706) **
Poor	3.439 (2.524-4.685) **	2.934 (2.138-4.027) **	2.856 (2.077-3.926) **
Present illness (ref; No)			
Yes	0.943 (0.811-1.097)	0.946 (0.813-1.101)	0.954 (0.820-1.111)
Instrumental activities of daily living (ref; Without difficulty)			
With difficulty	2.449 (2.167-2.767) **	2.287 (2.016-2.595) **	2.171 (1.911-2.467)
Alcohol consumption (ref; Non)			
Past	1.574 (1.005-2.467) *	1.550 (0.988-2.430)	1.624 (1.035-2.548) *

For peer review only

Current	0.915 (0.751-1.116)	0.936 (0.767-1.141)	0.949 (0.778-1.157)
**Smoking history (ref; Non-smoking)**			
Non-smoking now, quit before 5 years	1.490 (1.121-1.981) **	1.454 (1.093-1.934) *	1.455 (1.094-1.936) *
Non-smoking now, quit within 4 years	1.289 (0.758-2.193)	1.291 (0.759-2.197)	1.250 (0.735-2.129)
Smoking	1.468 (1.077-2.000) *	1.430 (1.048-1.949) *	1.400 (1.026-1.911) *
**Geriatric Depression Scale (ref; No depression)**			
Depression tendency		1.232 (1.074-1.414) **	1.240 (1.080-1.423) **
Depression		1.346 (1.116-1.624) **	1.364 (1.127-1.651) **
**Frequency of going outside (ref; Almost everyday)**			
One to three times a week		1.119 (0.985-1.272)	1.098 (0.965-1.249)
Once to twice a month or less		1.323 (1.120-1.563) **	1.257 (1.060-1.491) **
**Social isolation (ref, Non-isolation)**			
Moderately isolation			1.116 (0.977-1.275)
**Group participation in the community (ref; Non)**			
One			0.678 (0.555-0.829) **
Over two			0.736 (0.587-0.923) **
**Social support (ref; Non)**			
One			0.727 (0.408-1.295)
Over two			1.010 (0.619-1.647)
**Perception of community social cohesion (ref; Non)**			
One			0.912 (0.740-1.123)
Over two			1.145 (0.961-1.364)
Random effects			
**Community-level variance**	0.0202	0.0194	0.0209

*$p < 0.05$, **$p < 0.01$

Values presented are HRs (hazard ratios) and 95% CIs (confidence intervals). Community-level social capital variables (civic participation, social cohesion, and reciprocity) are 1 SD increase estimates. Variance of the intercept in the null model = 0.0351

For peer review only

For peer review only - http://bmjopen.bmj.com/site/about/guidelines.xhtml

DISCUSSION

To the best of our knowledge, this was the first study with a multilevel longitudinal design to examine the association between community social capital and the onset of functional disability using social capital indicators with verified validity in a large sample of older community-dwelling adults. The results suggested that living in a community with higher community social cohesion at baseline was associated with a lower future risk of functional disability, even after adjusting for individual responses on community social capital indicators. The present study indicated the importance of strategies to protect the health of older people through fostering cohesive communities with efforts such as promoting social connections and trust.

There are several possible pathways between community social cohesion and health. Social cohesion is determined by the resources available to members of tight-knit communities.[34] Cohesive communities might help residents to express trust toward their neighbors and to be psychologically healthier. Previous studies have revealed that neighborhood social cohesion positively affected older people's subjective well-being,[35, 36] and that cohesive communities prevented the occurrence of depressive symptoms in older people who lived alone and were at high risk of functional disability.[37] Thus, we considered high levels of community social cohesion to be potentially protective against

For peer review only

the onset of functional disability via the positive effects on psychological health, such as enhancing subjective well-being and inhibiting depressive symptoms.

Two previous studies examined the association between community social capital and the onset of functional disability in older people using multilevel longitudinal designs.[23, 24] Our results suggesting that higher community social cohesion was associated with lower risk of functional disability among men but not women were inconsistent with these previous studies. There are several possible reasons for this difference. First, both previous studies were surveys in a smaller area, compared with that in our research. Because our work used survey data from municipalities nationwide, the possibility for generalizing our findings might be higher. Second, the measurement index of community social capital in the previous studies differed from ours. Both previous studies used only one item ("general trust") to measure community social cohesion. In contrast, we used multidimensional indicators consisting of three measurement items with verified validity, which might be more accurate for examining community contextual effects. Therefore, our results might reflect more accurate estimates of the effects of community social cohesion on individual health.

In the present study, community social cohesion affected the onset of functional disability only among men. It is likely that, among people who are currently in the older

For peer review only - http://bmjopen.bmj.com/site/about/guidelines.xhtml

age groups, men sought and had stronger relationships with their colleagues before retirement than did women, given the nature of companies, particularly in Japan.[38] When this strong commitment is lost after retirement, these men may also experience a variety of changes in their living arrangements, leading to changes in their physical and mental health.[39] A cohesive community might be helpful in building new connections and encouraging social participation, which may keep men healthier and improve their psychological well-being. Honjo. et al. reported that rich social cohesion in a community buffered the risk of depression among older men living alone in Japan.[37] Thus, community cohesiveness may protect men's psychological health by helping them to build new connections in the community after retirement. However, further studies are needed to validate this hypothesis.

We considered urbanization (population density) a potentially confounding characteristic of living areas. In exploratory analyses, we confirmed that urbanization had a relatively strong influence as a confounding factor on the relationship between community social capital and the onset of functional disability. Therefore, the other characteristics of living area that were related to urbanization, such as public security, might have caused residual confounding. However, we believe that this influence was relatively small because we adjusted for urbanization as a representative factor of

For peer review only

communities.

The present study had several strengths. First, using a large, nationwide population-based sample enabled us to conduct a community-level multilevel analysis to clarify the contextual relationship between community-level social capital and the onset of functional disability. Second, we used validated indicators consisting of multidimensional items to measure community social capital. Therefore, we may have appropriately captured the whole of community social capital. However, the study also had several limitations. First, because the measurement was based on a self-administered questionnaire, the results are subject to response biases such as social desirability.[40] Social desirability bias may have artificially inflated social capital, which was calculated from the responses to the questionnaire. This, in turn, may have caused an overestimation of the relationship between community social capital and the onset of functional disability. Second, especially because the response rate to the survey was moderate (66.3%), selection bias might exist. Respondents in this study tended to be younger and healthier than the typical older adult population in the surveyed municipalities. In addition, people living in communities with low social capital might have been less likely than others to respond to the survey. These factors may have reduced the generalizability of our findings. However, because the respondents were

For peer review only - http://bmjopen.bmj.com/site/about/guidelines.xhtml

randomly selected or completely enumerated from 24 municipalities in Japan, we believe that any effect of selection bias was small. Third, there were frequently missing data on the model variables. In the analyses, we dealt with these missing data using a "missing" category." This approach had the potential to bias the results. Therefore, we conducted sensitivity analyses by removing the missing data (complete case analyses). These analyses confirmed that the tendencies of the results were almost the identical when the missing data were removed (data not shown). Fourth, our study included no information about changes in social capital. Therefore, it is possible that unmeasured time-varying covariates such as economic changes or natural disasters may have biased our results. Fifth, we used school district as the unit of analysis for communities because this was the smallest identifiable unit. However, the geographic scale of this unit may be slightly too large for the analysis of community-level social capital. Nevertheless, a school district represents an area of a size that older people can easily travel on foot or by bicycle, and community organizations, such as senior citizens' clubs and sports clubs, conduct their activities within individual school districts. Therefore, school district is a meaningful and appropriate unit of analysis for communities. Further work should build on our findings by defining regional units for spatial statistical analysis, using geographic information systems, for example. Finally, although our study was a prospective cohort

For peer review only - http://bmjopen.bmj.com/site/about/guidelines.xhtml

study, the follow-up period was moderately short. Considering the possibility of reverse causation, study designs with a longer follow-up period are necessary in the future.

CONCLUSION

In conclusion, this multilevel prospective cohort study found that higher levels of community social cohesion were associated with a lower incidence of onset of functional disability among older men, but not among older women, even after adjusting for individual social and behavioral variables. The findings suggest the importance of fostering cohesive communities to reduce the onset functional disability among older people.

ACKNOWLEDGMENTS

This study used data from the Japan Gerontological Evaluation Study (JAGES), conducted by the Center for Well-being and Society, Nihon Fukushi University, as one of their research projects. We thank Jennifer Barrett, PhD, from Edanz Group (www.edanzediting.com/ac) for editing a draft of this manuscript.

CONTRIBUTORS

For peer review only

TN had the idea for the study, participated in its design, performed the statistical analysis, and drafted the manuscript as the principal author. KK, the principal investigator of the JAGES project, helped to develop the idea of the study, participated in acquiring the data and in designing the study, and critically revised the manuscript. MS helped to develop the idea of the study, participated in acquiring the data and in designing the study, and critically revised the manuscript. HN helped with the data analysis and critically revised the manuscript. SS critically revised the manuscript. All authors read and approved the final manuscript.

FUNDING

This study used data from the Japan Gerontological Evaluation Study (JAGES), which was supported by the Ministry of Education, Culture, Sports, Science and Technology of Japan's Supported Program for the Strategic Research Foundation at Private Universities (2009–2013), the Japan Society for the Promotion of Science KAKENHI (Grant Numbers JP18390200, JP22330172, JP22390400, JP23243070, JP23590786, JP23790710, JP24390469, JP24530698, JP24683018, JP25253052, JP25870573, JP25870881, JP26285138, JP26882010, JP15H01972), Health Labour Sciences Research Grants (H22-Choju-Shitei-008, H24-Junkanki [Seishu]-Ippan-007, H24-

For peer review only

Chikyukibo-Ippan-009, H24-Choju-Wakate-009, H25-Kenki-Wakate-015, H25-Choju-Ippan-003, H26-Irryo-Shitei-003 [Fukkou], H26-Choju-Ippan-006, H27-Ninchisyou-Ippan-001), the Research and Development Grants for Longevity Science from Japan Agency for Medical Research and development (AMED) (JP18dk0110027, JP18ls0110002, JP18le0110009, JP19dk0110034), Research Funding for Longevity Sciences from the National Center for Geriatrics and Gerontology (24-17, 24-23, 29-42), and the World Health Organization Centre for Health Development (WHO Kobe Centre) (WHO APW 2017/713981). This work was supported by a grant from Nihon Fukushi University Research Institute for Health Sciences. The views and opinions expressed in this article are those of the authors and do not necessarily reflect the official policy or position of the funding organizations.

ETHICS APPROCAL

The ethics committee at Nihon Fukushi University approved the protocol and informed consent procedure for the present study (No. 10-05).

DATA SHARING

No additional data are available.

For peer review only - http://bmjopen.bmj.com/site/about/guidelines.xhtml

COMPERING INTERESTS

None declared.

REFERENCES

1 WHO. World Population Aging. New York: United Nations 2015.

2 International UaH. Aging in the Twenty-first Century: A Celebration and a Challenge. New York: UNFPA and HelpAge International 2012.

3 Ministry of Health, Labour and Welfare. Kaigo Hoken Jigyo Jyokyo Hokoku. Tokyo, Japan: Ministry of Health, Labour and Welfare 2010.

4 Keeler E, Guralnik JM, Tian H, et al. The impact of functional status on life expectancy in older persons. *J Gerontol A Biol Sci Med Sci* 2010;65A:727–33.

5 Putnam, RD. Making Democracy Work: Civic Traditions in Modern Italy. Princeton, NJ, USA: Princeton University Press 1993.

6 De Silva MJ, McKenzie K, Harpham T, et al. Social capital and mental illness: a systematic review. *J Epidemiol Community Health* 2005;59:619–27.

7 Murayama H, Fujiwara Y, Kawachi I. Social capital and health: a review of prospective multilevel studies. *J Epidemiol* 2012;22:179–87.

For peer review only - http://bmjopen.bmj.com/site/about/guidelines.xhtml

8 Story WT. Social capital and health in the least developed countries: a critical review of the literature and implications for a future research agenda. *Glob Public Health* 2013;8:983–99.

9 Nyqvist F, Forsman AK, Giuntoli G, et al. Social capital as a resource for mental well-being in older people: a systematic review. *Aging Ment Health* 2013;17:394–410.

10 McPherson KE, Kerr S, Morgan A, et al. The association between family and community social capital and health risk behaviours in young people: an integrative review. *BMC Public Health* 2013;13:971.

11 Nyqvist F, Pape B, Pellfolk T, et al. Structural and cognitive aspects of social capital and all-cause mortality: a meta-analysis of cohort studies. *Soc Indic Res* 2014;116:545–66.

12 Choi M, Mesa-Frias M, Nuesch E, et al. Social capital, mortality, cardiovascular events and cancer: a systematic review of prospective studies. *Int J Epidemiol* 2014;43:1895–1920.

13 Kawachi I, Subramanian SV, Kim D. Social capital and health: a decade of progress and beyond. In: Kawachi I, Subramanian SV, Kim D, eds. Social Capital and Health. New York: Springer 2008:1–26.

14 Wen M, Cagney KA, Christakis NA. Effect of specific aspects of community social

For peer review only

For peer review only - http://bmjopen.bmj.com/site/about/guidelines.xhtml

environment on the mortality of individuals diagnosed with serious illness. *Soc Sci Med* 2005;61:1119–34.

15 Mohan J, Twigg L, Barnard S, et al. Social capital, geography and health: a small-area analysis for England. *Soc Sci Med* 2005;60:1267–83.

16 Snelgrove JW, Pikhart H, Stafford M. A multilevel analysis of social capital and self-rated health: evidence from the British Household Panel Survey. *Soc Sci Med* 2009;68:1993–2001.

17 Oksanen T, Kouvonen A, Kivimäki M, et al. Social capital at work as a predictor of employee health: multilevel evidence from work units in Finland. *Soc Sci Med* 2008;66:637–49.

18 Desai RA, Dausey DJ, Rosenheck RA. Mental health service delivery and suicide risk: the role of individual patient and facility factors. *Am J Psychiatry* 2005;162:311–18.

19 Haseda M, Kondo N, Takagi D, et al. Community social capital and inequality in depressive symptoms among older Japanese adults: a multilevel study. *Health Place* 2018;52:8–17.

20 Yamaguchi M, Inoue Y, Shinozaki T, Saito M, Takagi D, Kondo K, Kondo N. Community social capital and depressive symptoms among older people in Japan: a multilevel longitudinal study. *J Epidemiol* (In press).

For peer review only - http://bmjopen.bmj.com/site/about/guidelines.xhtml

21 Koyama S, Aida J, Saito M, et al. Community social capital and tooth loss in Japanese older people: a longitudinal cohort study. *BMJ Open* 2016;6:e010768.

22 Aida J, Kondo K, Kawachi I, et al. Does social capital affect the incidence of functional disability in older Japanese? A prospective population-based cohort study. *J Epidemiol Community Health* 2013;67:42–7.

23 Imamura H, Hamano T, Michikawa T, et al. Relationships of community and individual level social capital with activities of daily living and death by gender. *Int J Environ Res Public Health* 2016;13:860.

24 Harpham T, Grant E, Thomas E. Measuring social capital within health surveys: key issues. *Health Policy Plan* 2002;17:106–11.

25 Baum FE, Ziersch AM. Social capital. *J Epidemiol Community Health* 2003;57:320–3.

26 Saito M, Kondo N, Aida J, et al. Development of an instrument for community-level health related social capital among Japanese older people: the JAGES Project. *J Epidemiol* 2017;27:221–7.

27 Kondo K. Progress in aging epidemiology in Japan: the JAGES Project. *J Epidemiol* 2016;26:331–6.

28 Tsutsui T, Muramatsu N. Care-needs certification in the long-term care insurance

For peer review only - http://bmjopen.bmj.com/site/about/guidelines.xhtml

system of Japan. *J Am Geriatr Soc* 2005;53:522–7.

29 Ministry of Health, Labour and Welfare. Long-term care, health and welfare services for the elderly. http://www.mhlw.go.jp/english/policy/care-welfare/care-welfare-elderly/index.html. Accessed 11 Nov 2018.

30 Matsuyama Y, Aida J, Watt RG, et al. Dental status and compression of life expectancy with disability. *J Dent Res* 2017;96:1006–13.

31 Koyano W, Shibata H, Nakazato K, et al. Measurement of competence: reliability and validity of the TMIG Index of Competence. *Arch Gerontol Geriatr* 1991;13:103–16.

32 Yesavage JA, Brink TL, Rose TL, et al. Development and validation of a geriatric depression screening scale: a preliminary report. *J Psychiatr Res* 1982;17:37–49.

33 Thabut G, Christie JD, Kremers WK, et al. Survival differences following lung transplantation among US transplant centers. *JAMA* 2010;304:53–60.

34 Kawachi I. Commentary: social capital and health: making the connections one step at a time. *Int J Epidemiol* 2006;35:989–93.

35 Cramm JM, Nieboer AP. Social cohesion and belonging predict the well-being of community-dwelling older people. *BMC Geriatr* 2015;15:30.

36 Cramm JM, van Dijk HM, Nieboer AP. The importance of neighborhood social cohesion and social capital for the well being of older adults in the community. *Tijdschr*

For peer review only

For peer review only - http://bmjopen.bmj.com/site/about/guidelines.xhtml

Gerontol Geriatr 2013;44:50–8.

37 Honjo K, Tani Y, Saito M, et al. Living alone or with others and depressive symptoms, and effect modification by residential social cohesion among older adults in Japan: the JAGES Longitudinal Study. *J Epidemiol* 2018;28:315–22.

38 Suzuki E, Takao S, Subramanian SV, et al. Work-based social networks and health status among Japanese employees. *J Epidemiol Community Health* 2009;63:692–6.

39 Oksanen T, Virtanen M. Health and retirement: a complex relationship. *Eur J Ageing* 2012;9:221–5.

40 Heitmann BL. Social desirability bias in dietary self-report may compromise the validity of dietary intake measures: implications for diet disease relationships. *Int J Epidemiol* 1996;25:222–5.

FIGURE LEGENDS

Figure 1. Flow chart showing participation in the study cohort, 2010 to 2013. LTCI: long-term care insurance; BADL: basic activity of daily living

For peer review only

For peer review only - http://bmjopen.bmj.com/site/about/guidelines.xhtml

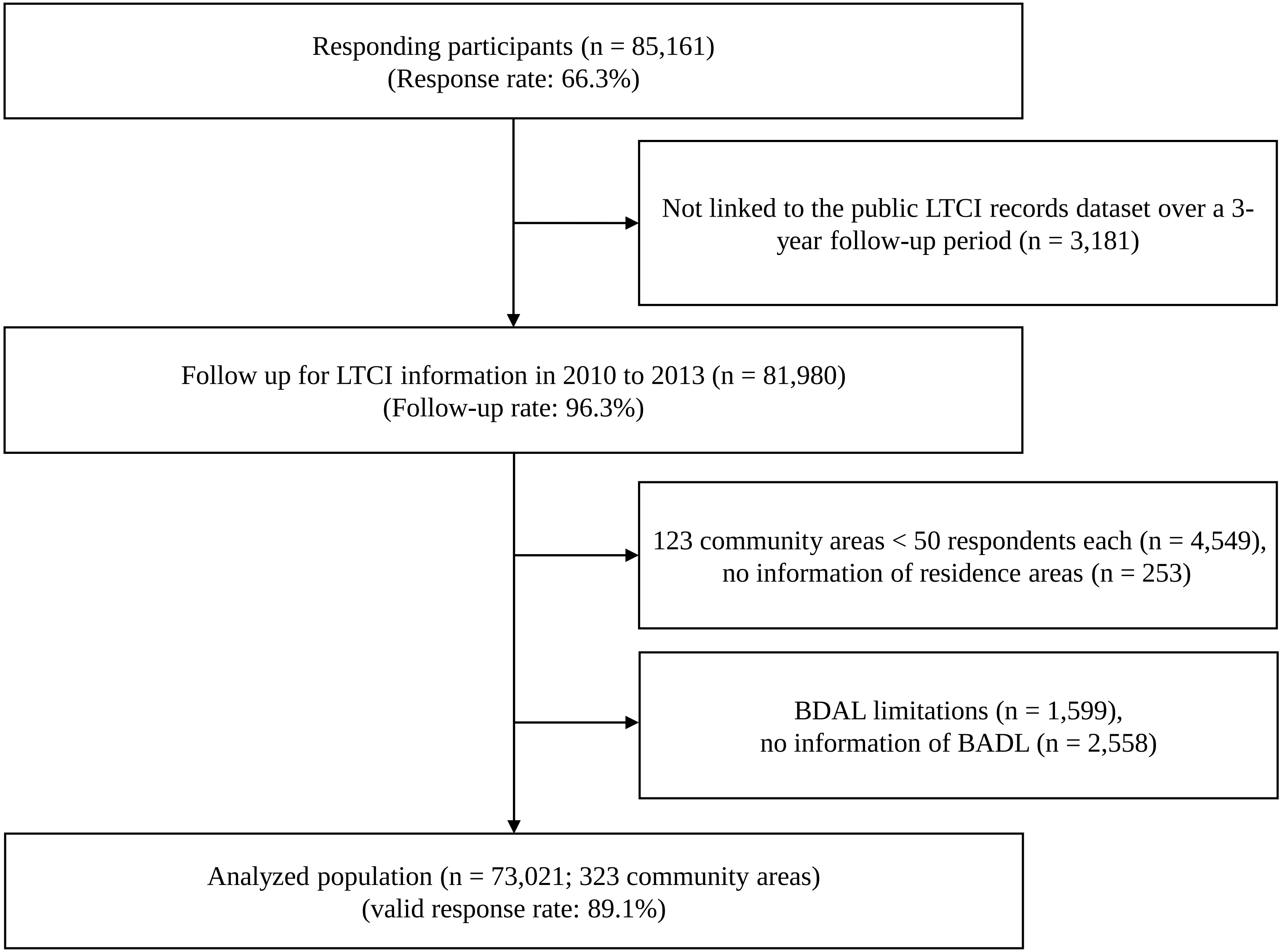
Responding participants (n = 85,161)
(Response rate: 66.3%)
Not linked to the public LTCI records dataset over a 3-year follow-up period (n = 3,181)
Follow up for LTCI information in 2010 to 2013 (n = 81,980)
(Follow-up rate: 96.3%)
123 community areas < 50 respondents each (n = 4,549),
no information of residence areas (n = 253)
BDAL limitations (n = 1,599),
no information of BADL (n = 2,558)
Analyzed population (n = 73,021; 323 community areas)
(valid response rate: 89.1%)

STROBE Statement—Checklist of items that should be included in reports of ***cohort studies***

	Item No	Recommendation	Page No
**Title and abstract**	1	(*a*) Indicate the study's design with a commonly used term in the title or the abstract	1
		(*b*) Provide in the abstract an informative and balanced summary of what was done and what was found	2-3
**Introduction**			
Background/rationale	2	Explain the scientific background and rationale for the investigation being reported	4-6
Objectives	3	State specific objectives, including any prespecified hypotheses	6
**Methods**			
Study design	4	Present key elements of study design early in the paper	6
Setting	5	Describe the setting, locations, and relevant dates, including periods of recruitment, exposure, follow-up, and data collection	6-7
Participants	6	(*a*) Give the eligibility criteria, and the sources and methods of selection of participants. Describe methods of follow-up	6-7
		(*b*) For matched studies, give matching criteria and number of exposed and unexposed	non
Variables	7	Clearly define all outcomes, exposures, predictors, potential confounders, and effect modifiers. Give diagnostic criteria, if applicable	7-12
Data sources/ measurement	8*	For each variable of interest, give sources of data and details of methods of assessment (measurement). Describe comparability of assessment methods if there is more than one group	7-12
Bias	9	Describe any efforts to address potential sources of bias	13-14
Study size	10	Explain how the study size was arrived at	7
Quantitative variables	11	Explain how quantitative variables were handled in the analyses. If applicable, describe which groupings were chosen and why	8-10, 11-12
Statistical methods	12	(*a*) Describe all statistical methods, including those used to control for confounding	12-14
		(*b*) Describe any methods used to examine subgroups and interactions	non
		(*c*) Explain how missing data were addressed	13-14
		(*d*) If applicable, explain how loss to follow-up was addressed	non
		(*e*) Describe any sensitivity analyses	non
**Results**			
Participants	13*	(a) Report numbers of individuals at each stage of study—eg numbers potentially eligible, examined for eligibility, confirmed eligible, included in the study, completing follow-up, and analysed	15
		(b) Give reasons for non-participation at each stage	7
		(c) Consider use of a flow diagram	7, figure 1
Descriptive data	14*	(a) Give characteristics of study participants (eg demographic, clinical, social) and information on exposures and potential confounders	15-18
		(b) Indicate number of participants with missing data for each variable of interest	15-18
		(c) Summarise follow-up time (eg, average and total amount)	15
Outcome data	15*	Report numbers of outcome events or summary measures over time	15

For peer review only

Main results	16	(*a*) Give unadjusted estimates and, if applicable, confounder-adjusted estimates and their precision (eg, 95% confidence interval). Make clear which confounders were adjusted for and why they were included	19-23
		(*b*) Report category boundaries when continuous variables were categorized	non
		(*c*) If relevant, consider translating estimates of relative risk into absolute risk for a meaningful time period	non
Other analyses	17	Report other analyses done—eg analyses of subgroups and interactions, and sensitivity analyses	non
**Discussion**			
Key results	18	Summarise key results with reference to study objectives	24
Limitations	19	Discuss limitations of the study, taking into account sources of potential bias or imprecision. Discuss both direction and magnitude of any potential bias	27-29
Interpretation	20	Give a cautious overall interpretation of results considering objectives, limitations, multiplicity of analyses, results from similar studies, and other relevant evidence	24-27
Generalisability	21	Discuss the generalisability (external validity) of the study results	25, 27
**Other information**			
Funding	22	Give the source of funding and the role of the funders for the present study and, if applicable, for the original study on which the present article is based	30-31

*Give information separately for exposed and unexposed groups.

Note: An Explanation and Elaboration article discusses each checklist item and gives methodological background and published examples of transparent reporting. The STROBE checklist is best used in conjunction with this article (freely available on the Web sites of PLoS Medicine at http://www.plosmedicine.org/, Annals of Internal Medicine at http://www.annals.org/, and Epidemiology at http://www.epidem.com/). Information on the STROBE Initiative is available at http://www.strobe-statement.org.

For peer review only
